# An Improved Grey Wolf Optimization with Multi-Strategy Ensemble for Robot Path Planning

**DOI:** 10.3390/s22186843

**Published:** 2022-09-09

**Authors:** Lin Dong, Xianfeng Yuan, Bingshuo Yan, Yong Song, Qingyang Xu, Xiongyan Yang

**Affiliations:** School of Mechanical, Electrical & Information Engineering, Shandong University, Weihai 264209, China

**Keywords:** grey wolf optimization, multi-strategy ensemble, exploitation and exploration, path planning

## Abstract

Grey wolf optimization (GWO) is a meta-heuristic algorithm inspired by the hierarchy and hunting behavior of grey wolves. GWO has the superiorities of simpler concept and fewer adjustment parameters, and has been widely used in different fields. However, there are some disadvantages in avoiding prematurity and falling into local optimum. This paper presents an improved grey wolf optimization (IGWO) to ameliorate these drawbacks. Firstly, a modified position update mechanism for pursuing high quality solutions is developed. By designing an ameliorative position update formula, a proper balance between the exploration and exploitation is achieved. Moreover, the leadership hierarchy is strengthened by proposing adaptive weights of *α*, *β* and *δ*. Then, a dynamic local optimum escape strategy is proposed to reinforce the ability of the algorithm to escape from the local stagnations. Finally, some individuals are repositioned with the aid of the positions of the leaders. These individuals are pulled to new positions near the leaders, helping to accelerate the convergence of the algorithm. To verify the effectiveness of IGWO, a series of contrast experiments are conducted. On the one hand, IGWO is compared with some state-of-the-art GWO variants and several promising meta-heuristic algorithms on 20 benchmark functions. Experimental results indicate that IGWO performs better than other competitors. On the other hand, the applicability of IGWO is verified by a robot global path planning problem, and simulation results demonstrate that IGWO can plan shorter and safer paths. Therefore, IGWO is successfully applied to the path planning as a new method.

## 1. Introduction

In recent years, with the development of robot technology, more and more work began to rely on robots to finish. Mobile robots gradually become a part of human industrial manufacture and mankind daily life [1,2,3,4]. Robots can execute operations such as perception and decision-making, assisting or even replacing human beings to complete heavy, repetitive or dangerous tasks. At present, the topics about robots covers robot navigation, robot mechanism design, human-robot interaction and so on [5,6,7,8]. One of the key technologies in robot navigation is global path planning [9,10], which refers to generating a safe and effective trajectory on the premise of knowing all environmental information. Path planning algorithms can be divided into traditional algorithms and meta-heuristic algorithms [11]. Traditional path planning algorithms include A* algorithm, rapidly-exploring random tree (RRT), Dijkstra algorithm, etc. Meta-heuristic algorithms include particle swarm optimization (PSO) [12], ant colony algorithm (ACO) [13], cuckoo algorithm (CS) [14], bat algorithm (BA) [15], grey wolf algorithm (GWO) [16], etc. On account of the advantages of fewer parameters and easy implementation, the investigation about meta-heuristic algorithms gains rapid expansion [17].

At present, heuristic algorithms have been widely applied. Wu et al. [18] successfully solved the graph partitioning problem using deterministic annealing neural network. In [19], Wu proposed a deterministic annealing neural network approach and verified the effectiveness of the algorithm through test problems. The meta-heuristic algorithm is regarded as an improved heuristic algorithm with the addition of random elements. Meta-heuristic algorithms, also known as intelligent optimization algorithms, are a class of methods that solve optimization problems based on computational intelligence mechanisms. They constantly imitate the rules, behaviors or mechanisms in physical, biological and social fields [20]. Due to the stochastic characters and the ability of saving the information in the optimization routine, meta-heuristic algorithms are feasible in settling complex and challenging optimization problems. In addition, meta-heuristic algorithms can be adapted to different applications by small adjustments [21]. Extensive researches and experiments in recent years indicate that meta-heuristic algorithms have unique advantages in solving robot path planning problems [22,23]. In [24], an improved ant colony algorithm was proposed and applied to the path planning of mobile robots. By extending the number of search directions of ants to 16, individual can query 24 neighborhoods of the current node each time, thus increasing the optional directions and search range in the search process and improving the search efficiency and accuracy of the algorithm. To enhance the ability of jumping out of the local minima, an improved PSO was proposed by employing an adaptive velocity formula into PSO [25]. Simultaneously, a robot path planning method with smoothing strategy was proposed by combining high-order Bezier curve. To improve the performance of PSO and solve the unmanned aerial vehicle (UAV) path planning problem in complex environment, spherical vector was introduced to PSO in [26]. Song et al. [27] designed a parallel cuckoo search algorithm (CS) to help UAV avoid obstacles and reach destination in a three-dimensional complex environment. In [28], a PSO variant based on evolutionary operator was proposed by introducing crossover operator and bee colony operator to solve multi-robot path planning problems. In [29], a hybrid PSO-GWO algorithm was proposed to optimize the robot trajectory, combining the characteristics of GWO and PSO.

GWO [16] was proposed by Seyedali Mirjalili et al. in 2014. This algorithm simulates the hunting process of grey wolves and has become one of the most popular algorithms in recent years [30,31]. GWO not only has the advantages of simple structure and few parameters like other meta-heuristic algorithms, but also possesses adaptive regulatory factor, which could better balance the intensification and diversification of GWO compared with other algorithms [32,33]. Therefore, GWO has attracted extensive attention of scholars and has been successfully applied in multifarious fields, including parameter extraction [34], job shop scheduling [35], feature selection [36], and disease classification prediction [37]. However, GWO also has the common problems of meta-heuristic algorithms, such as prematurity and slow convergence [38,39]. In order to improve the population diversity, reinforcement learning (RL) was introduced to GWO in [40]. Then the improved GWO was successfully applied to UAV path planning. In [41], aiming at the weakness that GWO is easy to fall into local optima in high-dimensional tasks, random leaders and random spiral-form motions were introduced into GWO. Heidari et al. [42] proposed an improved GWO based on Levy flight in order to cope with the disadvantages of prematurity and enhance the ability to solve global optimization problems. Luo [43] proposed an enhanced GWO variant to strengthen the search guidance of the head wolves. Experimental results proved that the convergence speed and accuracy of the algorithm were significantly improved. In [44], a new GWO variant was proposed for robot path planning, where the concept of loin swarm optimization (LSO) and dynamic weights were introduced into the original GWO to augment the searching ability of *α*, *β* and *δ* on the premise of ensuring population diversity. The above studies have manifested that GWO is useful in solving optimization problems, and the performance of the algorithm can be improved from different aspects.

In order to improve the performance of the algorithm, an improved grey wolf optimization (IGWO) is proposed in this paper, and its feasibility in the field of robot path planning is demonstrated by simulation experiments. It mainly possesses the three following improvements:

1. A modified position update mechanism is proposed to improve the accuracy of the solution by providing the algorithm with a better tradeoff of exploration and exploitation.

2. A dynamic local optimum escape strategy is designed, which can help the algorithm jump out of local optima traps when the algorithm is considered to be trapped in local stagnation.

3. An individual repositioning method is proposed. This strategy can pull back some individuals of the solutions to the vicinity of the current leaders compulsorily to accelerate the convergence of the algorithm in the anaphasis of the iterations.

To verify the performance of IGWO and the feasibility of IGWO to solve the path planning problem, this paper carries out extensive comparative experiments, including numerical optimization experiments and robot path planning examples. In the numerical optimization experiments, 20 benchmark functions are selected for two groups of statistical experiments. In the first set of experiments, IGWO is compared with five excellent GWO variants proposed recently. In the second group, seven well-established meta-heuristic algorithms including GWO are picked out in contrast with IGWO. Experimental results illustrate that IGWO has obvious advantages both in convergence accuracy and speed on the majority of benchmark functions. In robot path planning examples, this paper designs two obstacle environment models and fully verifies the applicability of IGWO with several comparison tests. In addition, cubic spline interpolation is used to smooth the path and obtain the path more aligned with the kinematic characteristics of robot. Experimental results exhibit that compared with the contrast algorithms, the routes planned by IGWO is not only shorter, but also more stable, indicating that IGWO has higher application value.

The remaining parts of the paper are structured as follows: Section 2 reviews the original GWO briefly. Section 3 provides a detailed description of the proposed approach. Section 4 illustrates the information of the benchmark functions used in experiments and analyzes the statistical results in detail, which proves that the proposed IGWO has better performance. The application of IGWO to settle robot path planning problems is highlighted in Section 5. The comparative test results of path planning are presented to verify the effectiveness and feasibility of the improved algorithm applied to path planning. Finally, Section 6 summarizes the conclusion and identifies future works.

## 2. Review of GWO

### 2.1. Leadership Hierarchy

**Figure 1:**
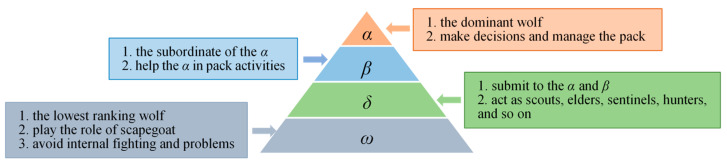
Leadership hierarchy.

Grey wolves live in a pack with strict social hierarchy, which is also one of the inspirations for GWO as shown in Figure 1. A grey wolf pack could be divided into 4 levels: alpha (*α*), beta (*β*), delta (*δ)* and omega (*ω*), from high to low, corresponding to the structural pyramid in Figure 1 from top to bottom. Each wolf plays specific role in the pack according to the level the wolf appertains to. *α*, also called the dominant wolf, is responsible for planning and deciding the hunting behavior of the group, representing the optimal solution obtained by the algorithm so far. *β* follows *α* in the rankings, whose duty is to assist *α* in decision-making. *δ*, ranking the third level in the social hierarchy, takes charge of reconnoitering, surveilling and guarding the pack as scouts, sentinels and guardians. Although *ω* wolves are in the lowest status of the tribe, their presence is essential to maintain the pack peace [16]. In the mathematical model of GWO, the first three best-fit solutions in the population are regarded as *α*, *β* and *δ* wolves, and the remaining wolves are regarded as *ω* wolves.

### 2.2. Hunting Mechanism

GWO not only mimics the leadership of grey wolves in nature, but also mimics the hunting mechanism [45]. The mathematical model is conducted with respect to the behavior of encircling prey, and the action mode can be described by Equations (1)–(5).

(1) $$D=\left|C\cdot X_{P}^{t}-X^{t}\right|$$

(2) $$X^{t+1}=X_{p}^{t}-A\cdot D$$

(3) $$A=2ar_{1}-a$$

(4) $$C=2r_{2}$$

(5) a=2−2(t/Max_iteration)

where *a* is the convergence factor, decreasing linearly from 2 to 0 with the number of iterations. *r*_1_ and *r*_2_ represent random vectors within [0, 1]. Both ***A*** and ***C*** are coefficient vectors. ***D*** denotes the approximate distance between the current wolf position and prey, ***X****^t^* and ***X****^t^*^+1^ stand for the positions of the wolf at *t*th and *t*+1th iteration respectively. $X_{p}^{t}$ represents the position of the prey at *t*th iteration.

**Figure 2:**
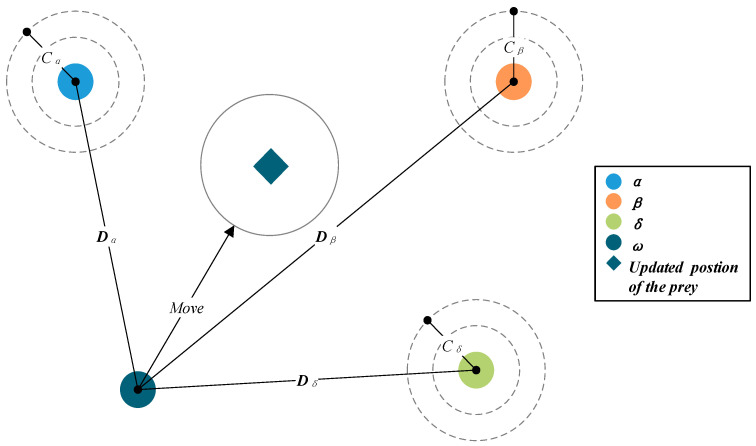
Update of position in GWO.

To mathematically describe the hunting mechanism, GWO assumes that the higher-level wolves are closer to the prey in the pack, that is, *α*, *β* and *δ* occupy the most powerful positions in the whole pack. Thus, the positions of remaining wolves are determined by the three wolves, which can be calculated as follows:

(6) $$\left\{ \begin{array}{l} D_{\alpha }=C_{1}\cdot X_{\alpha }^{t}-X^{t}\\ D_{\beta }=C_{2}\cdot X_{\beta }^{t}-X^{t}\\ D_{\delta }=C_{3}\cdot X_{\delta }^{t}-X^{t} \end{array}\right.$$

(7) $$\left\{ \begin{array}{l} X_{1}^{t}=X_{a}^{t}-A_{1}\cdot D_{\alpha }\\ X_{2}^{t}=X_{\beta }^{t}-A_{2}\cdot D_{\beta }\\ X_{3}^{t}=X_{\delta }^{t}-A_{3}\cdot D_{\delta } \end{array}\right.$$

(8) $$X^{t+1}=\frac{X_{1}^{t}+X_{2}^{t}+X_{3}^{t}}{3}$$

where ***C***_1_, ***C***_2_ and ***C***_3_ are random coefficient vectors defined by Equation (4). ***A***_1_, ***A***_2_ and ***A***_3_ are coefficient vectors defined by Equation (3). $X_{a}^{t}$, $X_{\beta }^{t}$ and $X_{\delta }^{t}$ represent the positions of *α*, *β* and *δ*. GWO pseudocode are shown in Algorithm 1, and the location update of wolves are exhibited in Figure 2.
**Algorithm 1.** The pseudocode of conventional GWO**1.** Generate a population ***X****_i_* (*i* = 1, 2, …, *n*) randomly **2.** Initialize the parameters of GWO (*max_iteration*, *a*, ***A*** and ***C***) **3.** Calculate the fitness values and assign *α*, *β* and *δ* **4. While** (*t* < *max_iteration*) **5.         For** each grey wolf **6.**                 Update the position of the current grey wolf using Equations (6)–(8) **7.         End for** **8.**         Update *a*, ***A*** and ***C*** **9.**         Amend the grey wolves’ positions beyond boundary limits **10.**       Calculate the fitness values of the new positions **11.**       Update the *α*, *β* and *δ* **12.**        *t* = *t* + 1 **13. End while** **14.** Return the position of *α*

## 3. Development of IGWO

**Figure 3:**
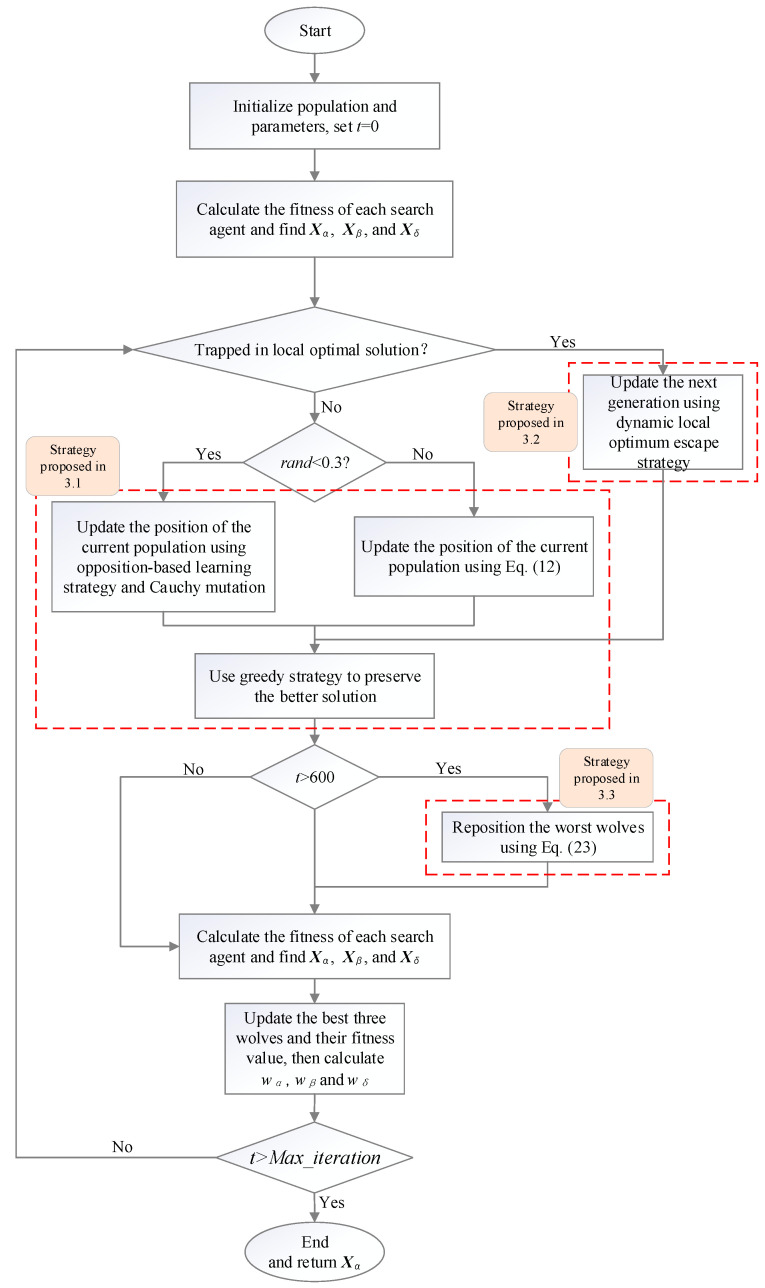
Framework of IGWO.

Three modifications are proposed in this section to overcome the defects of GWO. Firstly, a modified position update mechanism is designed, where an improved updating formula is constructed, and an enhanced diversity method including opposition-based learning strategy and Cauchy mutation is imported. In the proposed updating formula, the leaders (*α*, *β* and *δ*) compose the leader-determined term. The leaders will match different leadership authorities according to their fitness values, which is more suited to the hierarchy in nature. Furthermore, a random term is added on the basis of the leader-determined term to increase the exploration capability of the algorithm. Secondly, a dynamic local optimum escape strategy is proposed to escape from the local optimal value. In this strategy, a tuning parameter is employed to dynamically adjust the degree of variation. Finally, an individual repositioning method is imported to speed up the convergence rate of the algorithm by using the positions of *α*, *β* and *δ*. The framework of IGWO is presented in Figure 3 and the details of IGWO are introduced and discussed as follows.

### 3.1. Modified Position Update Mechanism

Wolves in nature have a strict hierarchy. The higher the rank of the wolf, the more dominant it is over the whole population and the stronger it is in the hunting process. A rigid hierarchy is crucial in the hunting process. However, in the hunting mechanism of GWO, as the position updating mechanism described by Equation (8), the leaders have the same power (1/3) over the population. It’s apparent that the distribution of power is incongruent with the real hierarchy.

**Figure 4:**
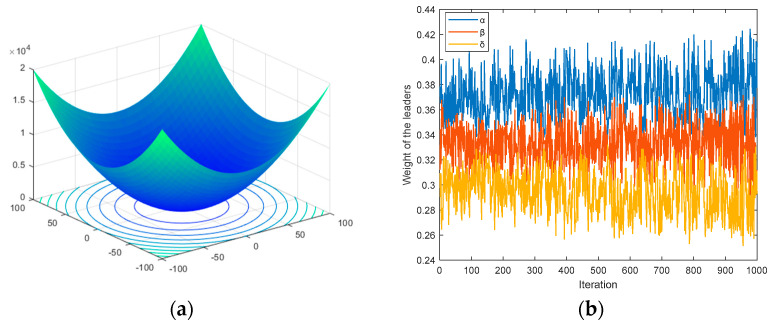
Adaptive weight coefficients of the leaders. (**a**) 2-D version of Sphere function; (**b**) Values of *w_α_*, *w_β_*, and *w_δ_*.

Due to the fitness values of the individuals can reflect the individuals’ merits and determine their hierarchy, this paper takes the fitness value into consideration and designs an adaptive weight coefficient based on the fitness value to better simulate the real hierarchy of the wolf pack. The mathematical expression is given as Equations (9)–(11).

(9) $$\theta_{i}=\left\{ \begin{array}{l} \frac{1}{\left|f\left(X_{i}\right)\right|+0.0001},\;i\in \left\{\alpha ,\;\beta ,\;\delta \right\},\;\mathrm{when}\;\mathrm{objective}\;\mathrm{function}\;\mathrm{is}\;\mathrm{minimized}\\ \left|f\left(X_{i}\right)\right|,\;i\in \left\{\alpha ,\;\beta ,\;\delta \right\},\;\mathrm{when}\;\mathrm{objective}\;\mathrm{function}\;\mathrm{is}\;\mathrm{maximized} \end{array}\right.\;$$

where $\theta_{i},\;i\in \left\{\alpha ,\;\beta ,\;\delta \right\}$ is the intermediate variable for calculating the adaptive weight coefficient. Moreover, the larger $\theta_{i}$ is, the closer the corresponding position is to the prey. In addition, the constant 0.0001 is added to prevent infinity when $f\left(X_{i}\right)$ equals 0 in the minimization problem. Then, the weight coefficients of α, β and δ can be calculated using Equation (10), and the position updating formula is rewritten as Equation (11).

(10) $$w_{i}=\theta_{i}/\left(\theta_{\alpha }+\theta_{\beta }+\theta_{\delta }\right),\;i\in \left\{\alpha ,\;\beta ,\;\delta \right\}\;$$

(11) $$X^{t}=rand\cdot \left(w_{\alpha }X_{\alpha }^{t}+w_{\beta }X_{\beta }^{t}+w_{\delta }X_{\delta }^{t}\right)$$

where *rand* is a random number between 0 and 1. Taking optimizing Sphere function as an example, Figure 4 shows the changing of the leaders’ weight coefficients (*w_α_*, *w_β_*, and *w_δ_*) calculated by Equations (9) and (10). It is obvious that during the optimization, the corresponding weights of the *α*, *β*, and *δ* are descend sequentially, which is more consistent with the hierarchical system of a wolf pack.

Because the individuals are randomly initialized and distributed in the search space, *α*, *β* and *δ* have similar probability of approaching prey compared to *ω* in preliminary phase. Apparently, the leader wolves are incompetent to guide the population search at the beginning. Hence using the leaders to guide the search would increase the risk of the premature convergence. To settle this deficiency, this paper adds a random term to the position update formula, which could randomly select individuals from the community to take charge of next search. Combining with Equation (11), the improved position updating formula is constructed ultimately as Equation (12).

(12) $$X^{t}=n_{1}\cdot rand_{2}\cdot \left(w_{\alpha }X_{1}^{t}+w_{\beta }X_{2}^{t}+w_{\delta }X_{3}^{t}\right)/3+n_{2}\cdot \left(X^{t}+rand_{2}\cdot \left(X_{rand}^{t}-X^{t}\right)\right)$$

(13) $$n_{1}=t/Max\_iteration$$

(14) $$n_{2}=1-n_{1}$$

where ***X****_t_*_+1_ and ***X****_t_* denote the current wolf’s position at *t+*1th and at *t*th iteration respectively. $X_{rand}^{t}$ represents the randomly selected wolf’s position. *rand*_1_ and *rand*_2_ are random numbers in [0, 1]. *n*_1_ and *n*_2_ are adjustment parameters that can bring about a tradeoff of the leader-determined term and the random term. Moreover, *n*_1_ and *n*_2_ are set to add up to 1, ensuring the convergence of solution. *n*_1_ will increase linearly to 1 with the number of iterations, and the corresponding search behavior could be described as follows: While *n*_1_ is smaller compared with *n*_2_ in the early stage, the random term provides the main guidance of movement for the pack, conducting a full exploration in search space. In the later stage, *n*_1_ increases to 1, and the dominating rights returns to the three leaders, enhancing the exploitation capacity of the approach. Compared with Equation (8), the new formula could better simulate the hierarchy of the wolf society, and balance the global and local searches.

In addition, this paper introduces the population opposition-based learning [46,47,48] and Cauchy mutation to increase population diversity. The mathematical model is shown in Equation (15), where *gamma* is the scale parameter of mutation. A threshold *τ* is set to decide how to renew the solutions. The enhanced diversity method will be executed with the probability of *τ* and Equation (12) will be used with the probability of (1 − *τ*). In this paper, *τ* is set to 0.3. Finally, the greedy strategy is employed in this paper to reserve the high-quality solutions. The mathematical expression is defined as Equation (16).

(15) $$X_{i}^{new}=\left\{ \begin{array}{l} X_{i}+Cauchy(o,gamma),\;i\in \left\{\alpha ,\;\beta ,\;\delta \right\}\\ rand(1,D)\cdot \left(ub+lb\right)-X_{i},\;else \end{array}\right.$$

(16) $$X^{t+1}=\left\{ \begin{array}{l} X^{t},\;if\;f\left(X^{t}\right)<f\left(X^{t+1}\right)\\ X^{t+1},\;otherwise \end{array}\right.\;$$

### 3.2. Dynamic Local Optimum Escape Strategy

The ultimate goal of this subsection is to address the concern of GWO being prone to fall into local minima. Inspired by random walk strategy in the improved symbiotic search algorithm (ISOS) [49], we design a dynamic local optimum escape strategy. The phenomenon of ISOS can be classified into three fundamental categories: mutualism phase, commensalism phase and parasitism phase. Previous researches point out that in the commensalism phase in nature, it is very likely that there are no suitable individuals nearby to establish commensalism relationship. It is crucial to explore new individuals and build commensalism. Miao [49] implemented a random walk strategy for the individual *i* to achieve a disturbance and jump out of the current solution. Then, the individual can be empowered to build commensalism relationship with the new result. More precisely, it’s the second component of Equation (17) that is the disturbance term to help the algorithm generate the new solution. And the specific model of random walk can be defined as follows:

(17) $$X_{new}=X+rand\left(1,\;D\right)\cdot \left(X_{m}^{t}-X_{n}^{t}\right)\cdot R_{i}$$

(18) $$R_{i}=\left\{ \begin{array}{l} 1,\;r>K\\ 0,\;otherwise \end{array}\right.$$

where *rand* (1, *D*) is a *D*-dimensional random matrix in the range of [0, 1].$\left(X_{m}^{t}-X_{n}^{t}\right)$ represents the distance of random walk.$X_{m}^{t},X_{n}^{t}$ are random candidates in the search space, satisfying *m* ≠ *n* ≠ *i*. *R**_i_*** is a logical value that determines whether to execute the random walk strategy. *K* is a fixed threshold. *r* is a random number. When *r* is greater than *K*, the random walk strategy will be executed. Otherwise, the original solution will be maintained.

In view of Cauchy mutation having large variation step, we propose dynamic local optimum escape strategy by importing the concept of Cauchy mutation. Its mathematical model can be described as follows:

(19) $$X^{t+1}=Cauchy\left(X^{t},\sigma \right)+rand_{3}\cdot X^{t}-rand_{4}\cdot X_{rand}^{t}$$

(20) $$\sigma =\frac{a}{2}\cdot \left|X_{\alpha }^{t}-X_{i}^{t}\right|$$

where *rand*_3_ and *rand*_4_ are random numbers inside [0, 1]. *σ* is a tuning parameter standing for the extent of mutation, which changes dynamically during the search process. *a* changes according to Equation (5). $rand_{3}\cdot X^{t}-rand_{4}\cdot X_{rand}^{t}$ is a small random disturbance that can further increase the ability of fleeing local optimum.

In addition, we select the top half of the superior agents as subgroup GS. The average fitness value of GS serves as the strategy’s enable switch. The average fitness value of GS can be calculated by Equation (21). The search state will be considered fallen into stagnation area and immediately activate dynamic local optimum escape strategy if Equation (22) is satisfied.

(21) $$mean\_GSvalue^{t}=\frac{2}{n}\sum_{i=1}^{n/2}f\left(X_{i}^{t}\right)$$

(22) $$mean\_GSvalue^{t+1}=mean\_GSvalue^{t}$$

In terms of the convergence of the algorithm, since the tuning parameter *σ* can be regulated according to the number of iterations and the distance from the predicted prey, it’s trustworthy that the proposed strategy does not affect the algorithm convergence. It is assumed that at the end of the evolutionary process, the average fitness of GS remains unchanged and *a* is close to 0. The whole pack is located near the prey in this period, causing $\left|X_{\alpha }^{t}-X_{i}^{t}\right|$ and $rand_{3}*X^{t}-rand_{4}*X_{rand}^{t}$ almost equaling to 0. It can be proved that the disturbance to the current position caused by the dynamic local optimum escape strategy is quite puny, guaranteeing the algorithm convergence.

### 3.3. Individual Repositioning Method

**Figure 5:**
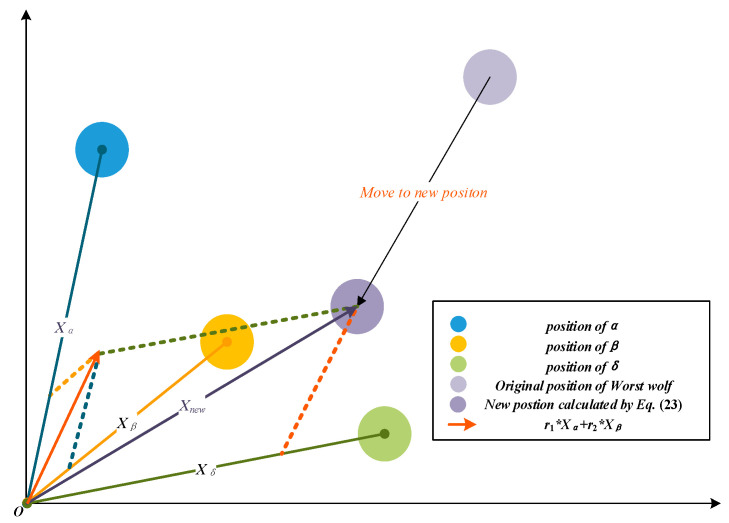
Reposition the worst wolves.

Miao et al. [50] repositioned the three wolves with the worst quality by using *α*, *β*, *δ* and the original positions in each iteration to remove the poor candidates and prevent search bias. Inspired by this idea, we propose a new individual repositioning method, which ignores the original position information and only uses the position of the head wolves, to speed up the convergence of the algorithm. The corrected position of the agents can be calculated by Equation (23), and the relocation process is shown in Figure 5.

(23) $$X_{new\_worst}=r_{1}\cdot X_{\alpha }+r_{2}\cdot X_{\beta }+r_{3}\cdot X_{\delta }$$

(24) $$r_{2}=rand(\frac{1-r_{1}}{2},1-r_{1})$$

(25) $$r_{3}=1-r_{1}-r_{2}$$

where ***X****_new_worst_* is the wolf’s position after repositioning, and *r*_1_ is a random number within [0.5, 1]. The sum of *r*_1_, *r*_2_ and *r*_3_ is set to 1 to ensure the convergence of the solutions.

The individuals that need to be relocated are those with low fitness. The purpose of the individual repositioning method is to make the search rapidly converge to the optimal value by correcting poorly behaved agents artificially. As the three leader wolves possess the best experience in the whole pack, it’s hoped that these individuals can be repositioned to the three leaders’ neighborhood, which is considered more likely to close to the prey. And the concept is embodied in Equation (23), where the positions of *α*, *β* and *δ* are used to generate a new position. Furthermore, as mentioned in Section 3.1, the authority ranking of the leaders is *α* > *β* > *δ*. To carry out the concept of the wolf hierarchy, the weights of the leaders are set to *r*_1_ > *r*_2_ > *r*_3_ in Equation (23).

It can be observed in Figure 5 that as the proposed individual repositioning method forces some individuals to relocate near the leaders, it can reduce the population diversity partly. However, the search prophase requires a high population diversity to find more potential optimal solutions. In other words, the individual repositioning method proposed in this subsection is supposed to be dormant in the early stage of search. Therefore, the position revising method won’t be activated until anaphase to maintain population diversity.

## 4. Numerical Optimization Experiments

In this section, the proposed algorithm is examined on 20 benchmark functions. These functions are all minimization problems, including 6 unimodal functions, 4 multimodal functions, 3 shifted and rotated multimodal functions, 4 fixed-dimension functions and 3 composite functions. The parameter settings of the functions are presented in Table 1 and Table 2, where *Dim* and *Range* represent the dimension and the boundary of the solution domain respectively, and F* symbolizes the optimal value for each benchmark function. To verify the superiority of IGWO, we conduct two suites of comparison experiments. In the first suite, IGWO is compared with five well-established GWO variants namely MixedGWO [51], GWOCS [34], LearnGWO [33], mGWO [52], and RW_GWO [53]. In the second suite, some mature meta-heuristic algorithms are chosen for the comparison of IGWO. These algorithms include GWO [16], particle swarm optimization (PSO) [12], artificial bee colony (ABC) [54], sine cosine algorithm (SCA) [55], whale optimization algorithm (WOA) [56], multi-verse optimizer (MVO) [57] and tunicate swarm algorithm (TSA) [58]. Additionally, to ensure the fairness and objectivity of the comparison experiments, all experiments are run on CPU Core I5-9400F and 8 GB RAM, and programmed on MATLAB R2018b.

### 4.1. Comparison of IGWO with Different GWO Variants

In this subsection, IGWO is compared with five state-of-the-art GWO variants. The number of iterations is set to 1000 and the population size is fixed to 30 for all algorithms. All algorithms run independently thirty times on each benchmark function to reduce the randomness of experimental results. Moreover, Wilcoxon rank-sum test at the significance level of 5% is adopted. “+”, “−” and “≈” respectively represent that IGWO is superior to, inferior to, and similar to the corresponding contrast method. The average value (Mean) and the standard deviation (Std) of the numerical results of 30 independent experiments are listed in Table 3 and Table 4, where the best Mean and Std are highlighted in bold. Meanwhile, Wilcoxon rank-sum test results (T) and algorithm ranking (R) are also recorded in Table 3 and Table 4. Finally, the overall Wilcoxon rank-sum test results and the mean ranking of the algorithms are provided in Table 5.

#### 4.1.1. Analysis of Numerical Results

Referring to the data presented in Table 3 and Table 4, IGWO hits the fittest results on fourteen benchmark functions out of twenty functions (14/20), then RW_GWO hits three out of twenty (3/20), then MixedGWO and LearnGWO hit two out of twenty (2/20). Finally, GWOCS and mGWO obtain zero out of twenty (0/22).

According to Table 3, with regard to unimodal functions (F1–F6), it can be proved that IGWO has higher chance of hitting the optimal value on most unimodal functions. It’s observed that IGWO ranks first on four functions (F1–F4) and obtains the theoretical optimal solutions of these benchmarks. Despite IGWO misses best result on F5 and F6, IGWO comes to the third place on F5 and the second place on F6 with the result very close to the best one. The improved updating formula can bring up an excellent tradeoff between exploitation and exploration, increasing the chance of positioning the optimum, while the greedy strategy ensures the preservation of the worthy candidates in each generation, which can further improve solution accuracy. For the multimodal functions (F7–F10), IGWO hits the global optimal values on F7 and F9 and obtains the best result among the six variants on F8, signifying the better search performance of the proposed IGWO. As the dynamic local optimum escape strategy can conduct more random operations, it can be easier for the algorithm to explore the search space more fully and avoid falling into the multiple local optimal peaks. For the shifted and rotated multimodal functions (F11–F13), IGWO gains best results on F12 and F13. Additionally, the results gained by RW_GWO are more accurate than remaining methods.

Table 4 indicates that, with respect to the multimodal fixed-dimension functions (F14–F17), IGWO yields the best results on F14 and F15, and locates the theoretical optimal values on F15 and F16. Meanwhile, MixedGWO converges to the theoretical optimal values on F16 and F17, and shows a little more stable search performance. For the composite functions (F18–F20), IGWO is superior to other approaches. Although LearnGWO get the same mean value as IGWO on F19 and F20, its standard deviation is bigger than that of IGWO, which means that IGWO is more stable and reliable than LearnGWO. The improvement of the performance should be owed to the modified strategies presented in this paper, which have diverse impact in dealing with different characteristics of the complex functions.

Furthermore, Table 5 summarizes the results above, showing the overall ranking of the six approaches is: IGWO > GWOCS > RW_GWO > LearnGWO > mGWO > MixedGWO.

#### 4.1.2. Analysis of Convergence Curves

**Figure 6:**
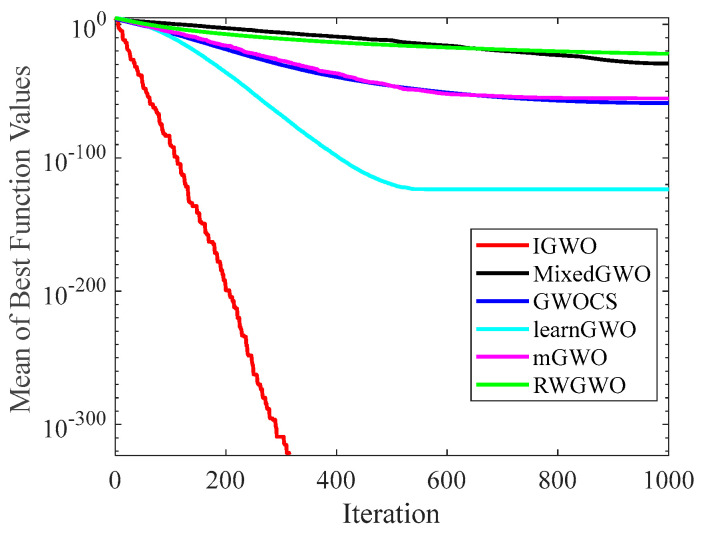
Comparison results of GWO variants on F1.

**Figure 7:**
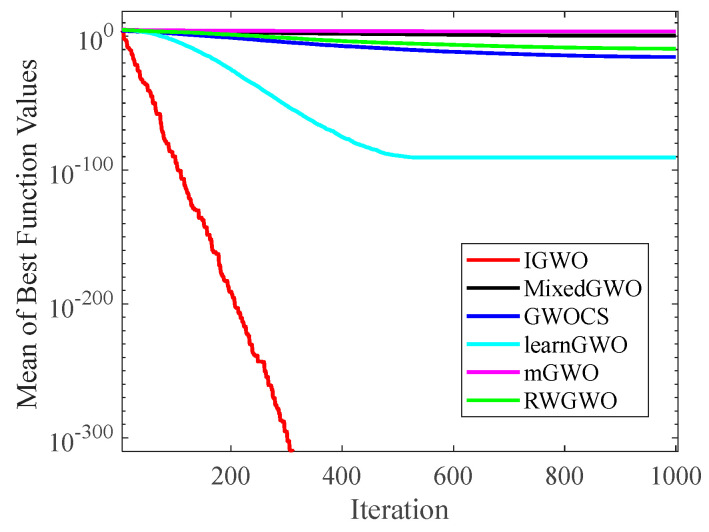
Comparison results of GWO variants on F3.

**Figure 8:**
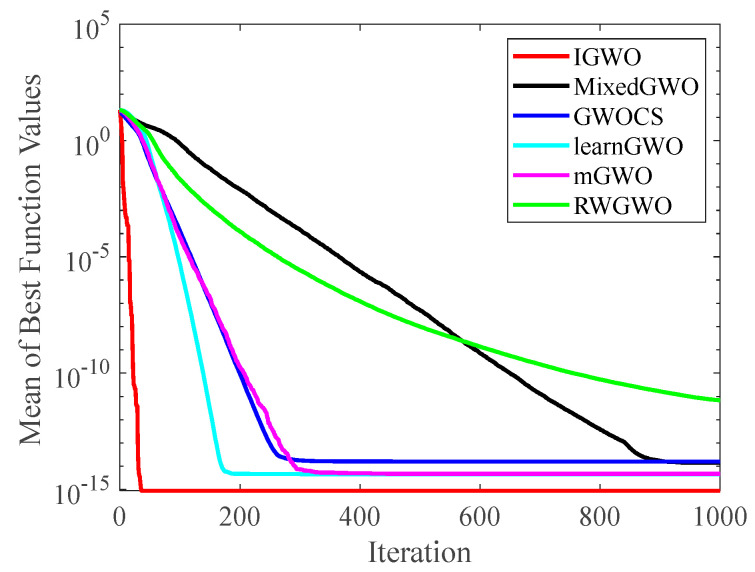
Comparison results of GWO variants on F8.

**Figure 9:**
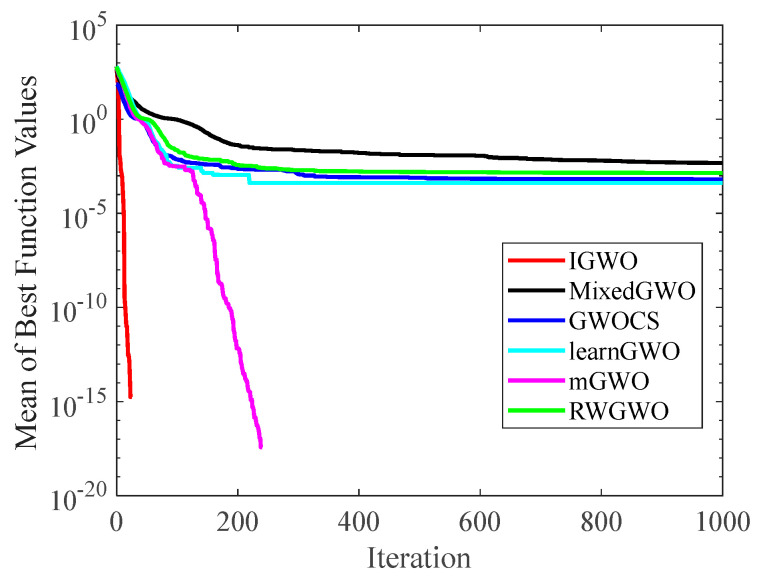
Comparison results of GWO variants on F9.

**Figure 10:**
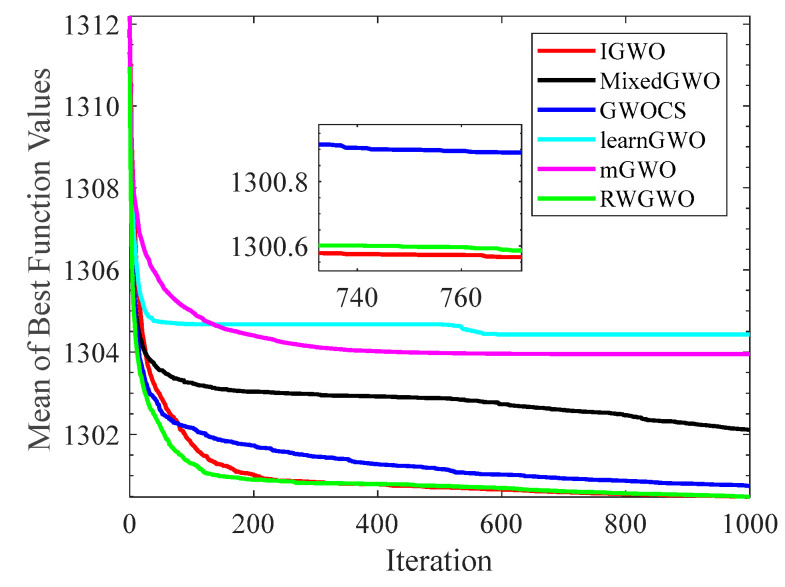
Comparison results of GWO variants on F12.

**Figure 11:**
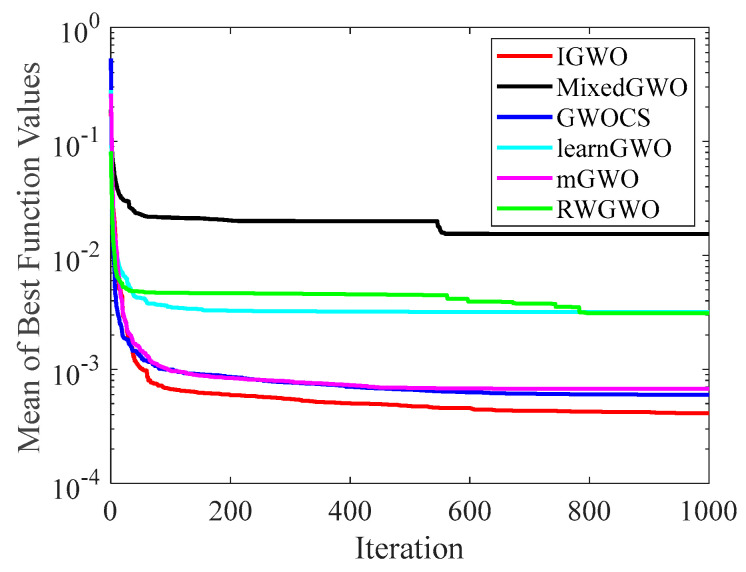
Comparison results of GWO variants on F14.

**Figure 12:**
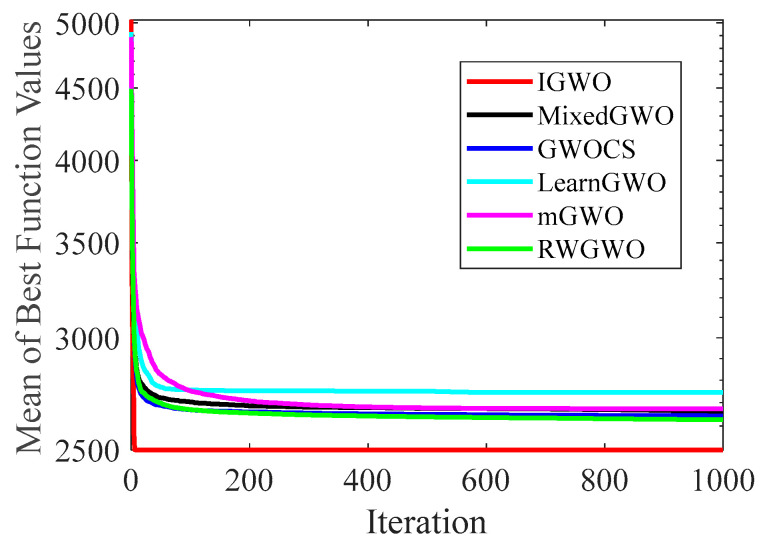
Comparison results of GWO variants on F18.

**Figure 13:**
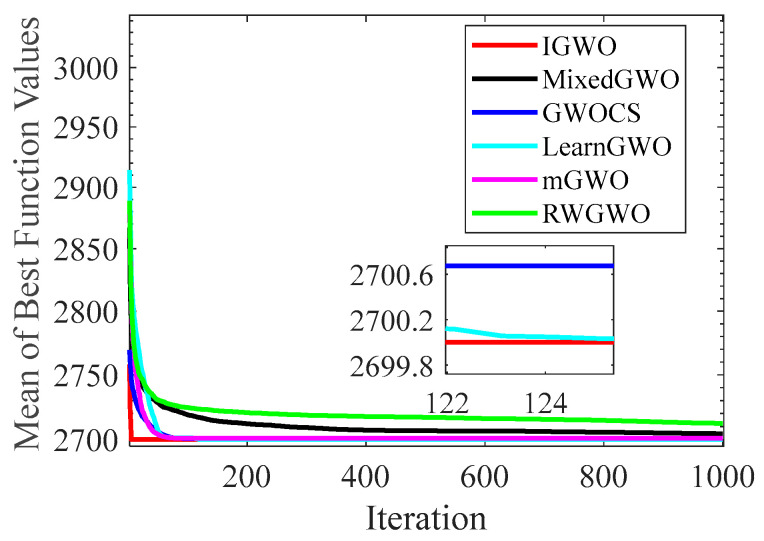
Comparison results of GWO variants on F20.

Figure 6, Figure 7, Figure 8, Figure 9, Figure 10, Figure 11, Figure 12 and Figure 13 exhibit the convergence curves of the six GWO variants on eight test functions (F1, F3, F8, F9, F12, F14, F18, and F20). The *x*-axis and the *y*-axis represent the iterations and the corresponding best average fitness values achieved by thirty independent experiments, respectively.

Figure 6 and Figure 7 indicates that the curve of IGWO can converge to the global optimum at a gallop, confirming that IGWO has better search accuracy and speed on F1 and F3. Furthermore, LearnGWO performed relatively well on F1 and F3 in comparison to MixedGWO, GWOCS, RW_GWO and mGWO, with the accuracy up to 1E10-120. Figure 8 illustrates that after about forty iterations, IGWO has already reached the final result with higher accuracy. From Figure 9, it can be observed that both IGWO and mGWO can converge to the optimal value on F9 rapidly, but IGWO can avoid the local optimum more efficiently. Figure 10 shows that RW_GWO and IGWO own higher convergence accuracy on F12, and IGWO ranks first by virtue of slightly higher accuracy. For the function F14, Figure 11 exhibits that the convergence speed of the six algorithms is similar, but the convergence accuracy of IGWO is slightly better. As shown in Figure 12 and Figure 13, compared with other improved versions, IGWO has noticeable virtue both in convergence speed and accuracy. In addition, Figure 13 demonstrates that the accuracy gap of the six algorithms is small, yet IGWO possesses an obvious advantage in the search speed. In summary, as presented in Figure 6, Figure 7, Figure 8, Figure 9, Figure 10, Figure 11, Figure 12 and Figure 13, the proposed strategies can visually improve the search accuracy and convergence speed on most functions.

Therefore, from the above discussions of Table 3, Table 4 and Table 5 and Figure 6, Figure 7, Figure 8, Figure 9, Figure 10, Figure 11, Figure 12 and Figure 13, it can be concluded that the proposed IGWO has better optimization precision and convergence speed for the majority of the twenty benchmarks, which indicates that the proposed strategies can effectively improve the performance of the algorithm.

### 4.2. Comparison of IGWO with Other Meta-Heuristic Algorithms

In this subsection, IGWO is compared with the original GWO and six other well-known meta-heuristic algorithms, including PSO, ABC, SCA, WOA, MVO and TSA. The benchmark functions used in this suite are enumerated in Table 1 and Table 2. Each algorithm runs independently 30 times, and the average value (Mean) and the standard deviation (Std) of the results are recorded in Table 6 and Table 7. The optimal Mean and standard deviation obtained by the eight algorithms are marked in bold. In addition, Wilcoxon rank-sum test is also conducted with the confidence interval of 5% to test whether there are significant differences between the proposed variant and other competitors, and the algorithms are ranked. The rank-sum test results (T) and the final ranking results (R) are shown in Table 6 and Table 7. Table 8 summarizes the data above.

#### 4.2.1. Analysis of Numerical Results

Referring to the results presented in Table 6 and Table 7, IGWO hits the best results on thirteen benchmark functions out of twenty functions (13/20), then TSA both hit three out of twenty (3/20), then PSO and ABC hits two out of twenty (2/20), then GWO and WOA hit one out of twenty (1/20). Finally, SCA and MVO obtain zero out of twenty (0/22). As shown in Table 6, IGWO overtakes all the comparison algorithms on unimodal functions except F5. Particularly, IGWO reaches the theoretical optimal values on F1–F4. For the multimodal functions (F7–F10), IGWO beats other contrast meta-heuristic algorithms on F7–F9, and converges to the theoretical optimal values on F7 and F9. For F8, compared with GWO, the search accuracy of IGWO is improved by 2 orders of magnitude. With respect to the shifted and rotated multimodal functions (F11–F13), IGWO obtains the best result among other meta-heuristic algorithms on F12. PSO and TSA do best on F11 and F13 respectively, whereas IGWO ranks third on F11 and second on F13, and the results are close to the best.

For fixed-dimension functions (F14–F17), IGWO obtains the best result on F14 and reaches the theoretical optimal values on F15 and F16. TSA ranks the first on F17, but the result gained by IGWO is also in good quality. The results acquired by IGWO are closer to the optimal solution on the composite functions (F18–F20), and the search performance is more stable by referring to the corresponding Std. Besides, compared with the original GWO, IGWO shows competitive performance except for F5 and F10, which is inseparable with the strategies proposed in this paper.

Table 8 exhibits the overall Wilcoxon rank-sum test results and average ranking of the algorithms according to Table 6 and Table 7. The average ranking of IGWO is 1.7, manifesting that IGWO has outstanding search ability in function optimization problems compared with the other seven algorithms. Furthermore, we can reach the conclusion that the performance ranking of the algorithms in this part is IGWO > GWO > WOA > PSO > TSA > MVO > ABC > SCA.

#### 4.2.2. Analysis of Convergence Curve

**Figure 14:**
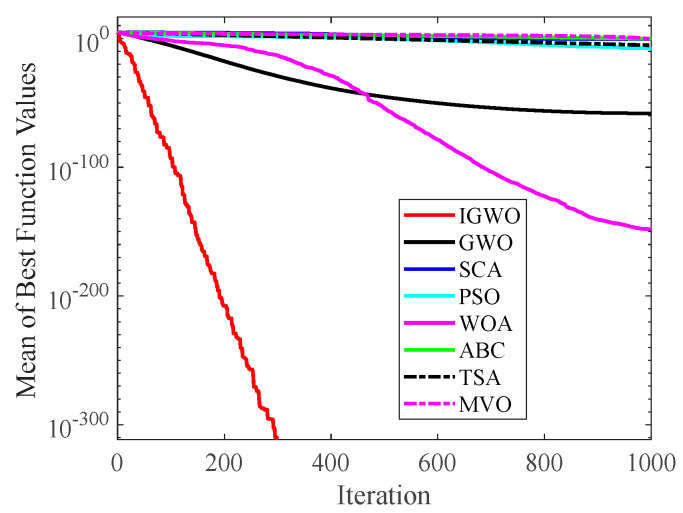
Comparison results on F1.

**Figure 15:**
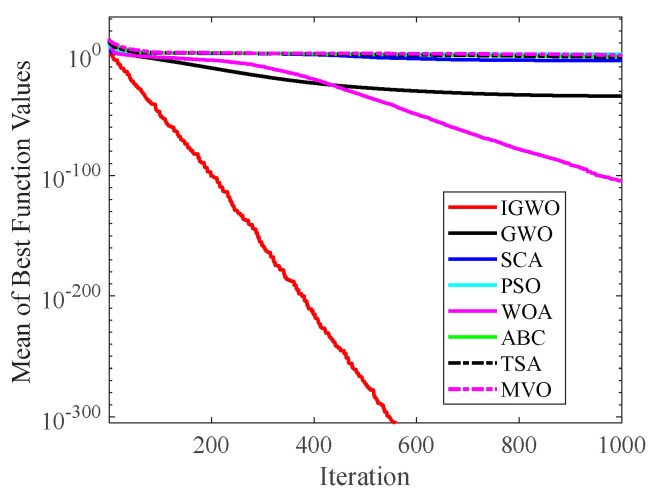
Comparison results on F2.

**Figure 16:**
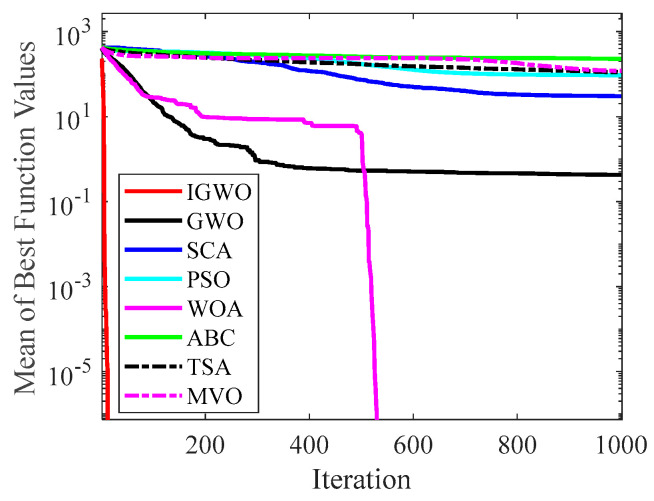
Comparison results on F7.

**Figure 17:**
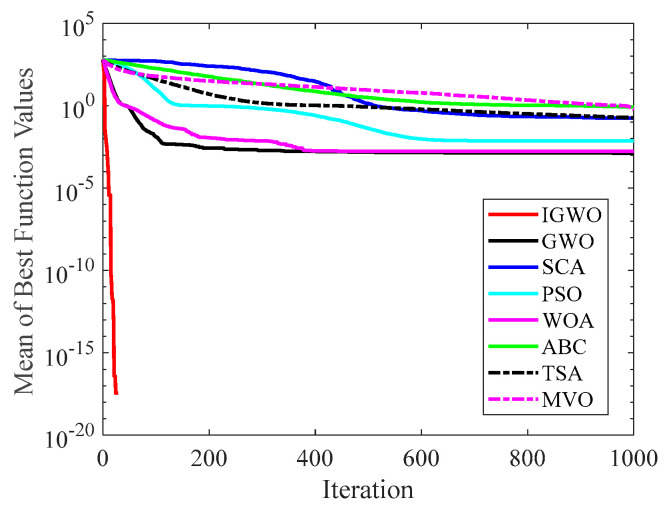
Comparison results on F9.

**Figure 18:**
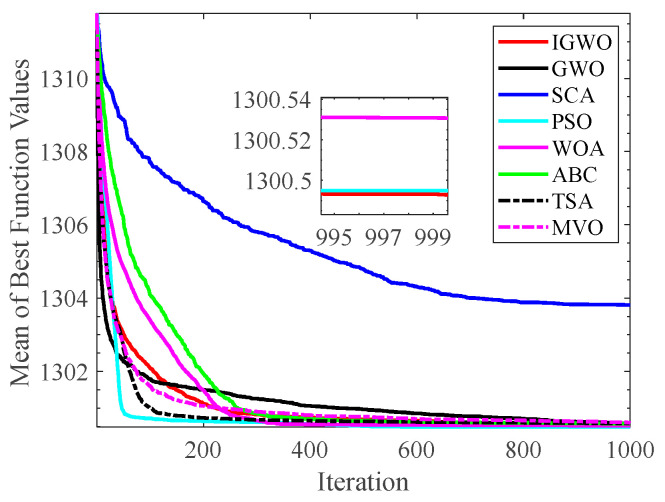
Comparison results on F12.

**Figure 19:**
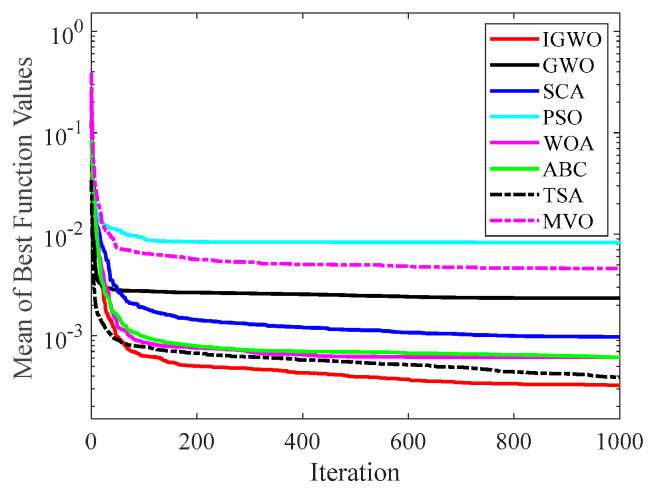
Comparison results on F14.

**Figure 20:**
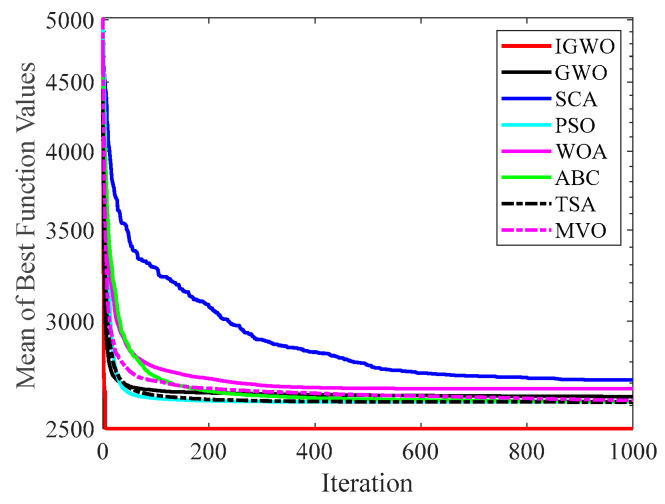
Comparison results on F18.

**Figure 21:**
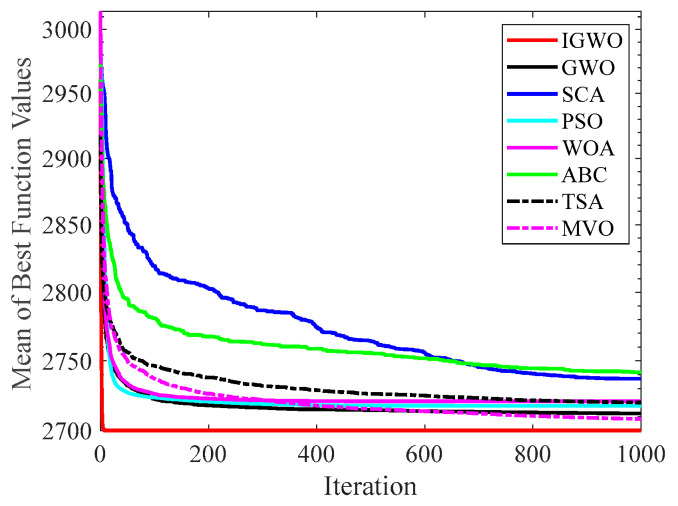
Comparison results on F20.

Figure 14, Figure 15, Figure 16, Figure 17, Figure 18, Figure 19, Figure 20 and Figure 21 illustrate the convergence curves of IGWO and seven contrast algorithms on partial functions (F1, F2, F7, F9, F12, F14, F18 and F20). The number of iterations of each algorithm is set to 1000, and the value corresponding to each iteration in the curve is the mean value of thirty independent experimental results.

Figure 14 and Figure 15 indicate that IGWO outperforms all other methods on F1 and F2, where IGWO can converge to the global optimal value at the fastest speed. Meanwhile, WOA reaches the second place by means of faster convergence speed and higher accuracy on F1 and F2. Figure 16 reveals that the performance of IGWO is significantly better than others on F7, and WOA achieves similar search accuracy to IGWO. It’s worth mentioning that WOA traps into the local minimums during the 200th iteration to the 500th while IGWO converges rapidly. Therefore, IGWO algorithm has better local optimum avoidance ability. The preeminence of IGWO can be noted in Figure 17 in terms of the search accuracy and speed on multimodal function F9. Figure 18 shows that all algorithms obtain similar results and IGWO obtains slightly better result on F12. As shown in Figure 19, IGWO and TSA all achieve better results on F14, while the result obtained by IGWO owns slightly higher accuracy. Figure 20 and Figure 21 indicate that solution accuracy gained by IGWO ranks first on the composite functions F18 and F20, with the obvious speed advantage contemporarily. Figure 14, Figure 15, Figure 16, Figure 17, Figure 18, Figure 19, Figure 20 and Figure 21 vividly show the search performance of the eight methods, certifying that the proposed IGWO has excellent performance on most functions.

## 5. Application of IGWO in Robot Path Planning

To verify the feasibility of IGWO to solve the robot path planning problem, this section designs two terrains to simulate the environment where the robot works, one is simple and the other is complex. The proposed IGWO is compared with MEGWO [59] and RMPSO [60] on the experiments. In addition, we introduce cubic spline interpolation to smooth the planned track, making it more in line with the dynamics characteristics of robots and increasing application value.

### 5.1. Environment Models

**Figure 22:**
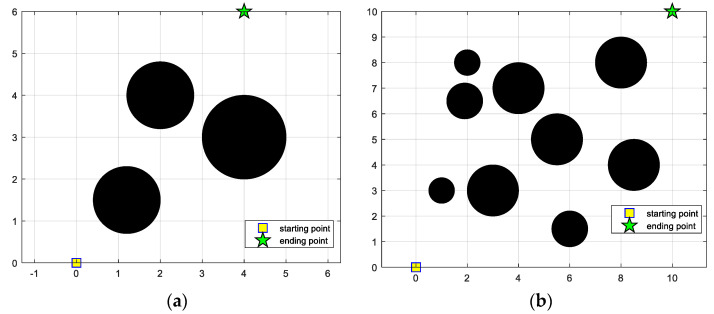
Basic environment models. (**a**) Simple obstacle environment model (case 1); (**b**) Complex obstacle environment model (case 2).

To simplify the problem, we make two assumptions: (a) Since the area of a circle is larger than that of a square when the perimeters are equal, the obstacle is set as a circle; (b) The size of the robot is added to the radius of the obstacle, so that the robot can be regarded as a mass point. Based on the above assumptions, the two kinds of environmental models are designed as shown in Figure 22. In the environmental models, the starting points, ending points and obstacles are represented by yellow squares, green pentagrams and black circles, respectively. The simple obstacle environment model consists of three obstacles, with the starting point set as (0, 0) and the end point set as (4, 6), while the complex environment model contains nine obstacles, with the starting point at (0, 0) and the end point at (10, 10). Additionally, the Euclidean distance between the beginning and the end is considered to be the shortest path length. The information of the two terrains is listed in Table 9. The mathematical expression of the obstacle in the coordinate system is defined as follows:

(26) $$r^{2}={\left(x-x_{obs}\right)}^{2}+{\left(y-y_{obs}\right)}^{2}$$

where *r* is the radius of the obstacle, and (*x_obs_*, *y_obs_*) represents the coordinates of the center of the obstacle.

### 5.2. Path Smoothing

Wang et al. [61] smoothed the planned path by utilizing inner arcs to ensure the continuity of robot motion. Liu et al. [62] pointed out that using the cubic spline interpolation to smooth path has significant virtues compared with circular arcs or straight line and less constraints compared with fillet fitting. Therefore, we adopt the cubic spline interpolation to smooth the trajectory. Cubic spline interpolation is a piecewise interpolation method, which obtains a smooth curve through a series of interpolation points based on cubic polynomials. The interpolation process can be described as follows:

Take *n* + 1 nodes on a given interval [a, b], then the interval can be divided into *n* subintervals $\left[\left(x_{0},\;x_{1}\right),\left(x_{1},\;x_{2}\right),\ldots ,\left(x_{n-1},\;x_{n}\right)\right]$, namely segmentation process. And the value of the node is given in advance as follows:

(27) $$f\left(x_{i}\right)=f_{i},\;i=0,1,2,\ldots ,n$$

Suppose there is a function *S*(*x*), if *S*(*x*) satisfies the following conditions:

1. $S\left(x\right),S^{\prime }\left(x\right),S^{\prime \prime }\left(x\right)$ is continuous.

2. $S\left(x_{i}\right)=f_{i},\;i=0,1,2,\ldots ,n$

3. $S\left(x\right)=S_{i}\left(x\right),\;x\in \left[x_{i},\;x_{i+1}\right]$ is a cubic polynomial.

Then *S*(*x*) is called a cubic spline interpolation function, whose curve can be utilized to generate the required curve. Furthermore, on each subinterval [*x_i_*, *x_i_*_+1_], *S*(*x*) is defined as follows:

(28) $$S\left(x\right)=a_{i}x^{3}+b_{i}x^{2}+c_{i}x+d_{i},\;i=0,1,2,\ldots ,n-1$$

where *a_i_*, *b_i_*, *c_i_* and *d_i_* are undetermined coefficients. As *S*(*x*) has 4*n* undetermined coefficients, there is a need for at least 4*n* known conditions to solve the undetermined coefficients, and the specific conditions are given as follows:

*n* + 1: $S\left(x_{i}\right)=f_{i},\;i=0,1,2,\ldots ,n$

*n* − 1: ${S^{\prime }}_{-}\left(x_{i}\right)={S^{\prime }}_{+}\left(x_{i}\right),\;i=1,2,\ldots ,n-1$

*n* − 1: ${S^{\prime \prime }}_{-}\left(x_{i}\right)={S^{\prime \prime }}_{+}\left(x_{i}\right),\;i=1,2,\ldots ,n-1$

The other two conditions can be obtained by boundary conditions. The commonly used three boundary conditions can be described as follows:

1. Clamped Spline: $S^{\prime }\left(x_{0}\right)=A,\;S^{\prime }\left(x_{n}\right)=B$, where *A* and *B* are specified.

2. Natural Spline: $S^{\prime \prime }\left(x_{0}\right)=S^{\prime \prime }\left(x_{n}\right)=0$.

3. Not-A-Knot Spline: $S^{\prime \prime \prime }\left(x_{0}\right)=S^{\prime \prime \prime }\left(x_{1}\right),\;S^{\prime \prime \prime }\left(x_{n-1}\right)=S^{\prime \prime \prime }\left(x_{n}\right)$.

The proposed IGWO is applied to robot path planning problems. The common points between adjacent subintervals are considered as path nodes, and each node represents a turn along the planned path. Thus, we take the number of path nodes as the dimension of a grey wolf, that is, an individual of the wolf pack represents a candidate path.

Suppose *D* path nodes are given, and their coordinates are (*x_dD_*, *y_dD_*), (*x_dD_*, *y_dD_*), …, (*x_dD_*, *y_dD_*), where the starting and ending coordinates are (*x_s_*, *y_s_*) and (*x_t_*, *y_t_*). Firstly, split the abscissa and ordinate of the above *D* + 2 points into the sets of {*w_x_*} = {*x_s_*, *x_d_*_1_, *x_d_*_2_,…, *x_dD_*, *x_t_*} and {*w_y_*} = {*y_s_*, *y_d_*_1_, *y_d_*_2_,…, *y_dD_*, *y_t_*}. Then, apply cubic spline interpolation to{*w_x_*} and {*w_y_*} separately and obtain the abscissa and the ordinate of *n* interpolation points, namely {*x*_1_, *x*_2_,…, *x_n_*} and { *y*_1_, *y*_2_,…, *y_n_*}. Finally, {(*x_s_*, *y_s_*), (*x*_1_, *y*_1_), (*x*_2_, *y*_2_), …, (*x_n_*, *y_n_*), (*x_t_*, *y_t_*)} is the path of the robot after smoothing.

### 5.3. Construction of Fitness Function

The purpose of robot path planning is to plan a shortest path without collision with obstacles from the starting point (*x_s_*, *y_s_*) to the end point (*x_t_*, *y_t_*) [63]. Therefore, a fitness function is constructed to measure the performance of a robot obstacle avoidance and path length in this subsection. The mathematical model is defined as follows:

(29) $$f=f_{l}*\left(1+\lambda *f_{obs}\right)$$

where *λ* is a penalty factor employed to exclude the candidates through the obstacle area. *f_l_* is the sum of Euclidean distance between the interpolation points and the calculation method is shown in Equation (30). *f_obs_* is a marker variable to evaluate the path obstacle avoidance level, whose initial value is 0. *f_obs_* can be calculated using Equations (31)–(33).

(30) $$f_{l}=\sum_{i=1}^{n}\sqrt{{\left(x_{i+1}-x_{i}\right)}^{2}+{\left(y_{i+1}-y_{i}\right)}^{2}}$$

(31) $$d_{k}^{i}=\sqrt{{\left(x_{i}-x\_obs_{k}\right)}^{2}+{\left(y_{i}-y\_obs_{k}\right)}^{2}},\;i=1,2,\ldots ,n,\;k=1,2,\ldots ,n_{obs}$$

(32) $$\varepsilon_{k}=\frac{1}{n}\sum_{i=1}^{n}MAX\left(1-d_{k}^{i}/r_{k},\;0\right),\;k=1,2,\ldots ,n_{obs}$$

(33) $$f_{obs}=\sum_{k=1}^{n_{obs}}\varepsilon_{k}$$

where *x_i_* and *y_i_* respectively represent the *x*-coordinate and *y*-coordinate of the *i*^th^ interpolation point on a path. (*x*_*obs_k_*, *y*_*obs_k_*) is on behalf of the center coordinate of the *k*^th^ obstacle. *n* is the number of interpolation points of the path. *n_obs_* is the total number of obstacles and *r_k_* is the corresponding radius of the obstacle. Equation (31) is to deal with the Euclidean distance between the interpolation point on the candidate path and the center of *k*^th^ obstacle. Equation (32) could determine whether the path intersects with the *k*^th^ obstacle. If there is a path point entering the *k*^th^ obstacle, then *ε_k_* > 0. Otherwise, *ε_k_* = 0. Moreover, if a planned path avoids all the obstacles successfully, *f_obs_* = 0.

### 5.4. Experimental Environment and Parameter Setting

To pursue the objective and fair experimental results, RMPSO, MEGWO and IGWO all use the same software and hardware platform. The population size, the number of path nodes (i.e., individual dimension), number of interpolation points, and the maximum of iterations of the three algorithms remain consistent, as shown in Table 10.

### 5.5. Analysis of Path Planning Results

#### 5.5.1. Single Contrast Experiment

**Figure 23:**
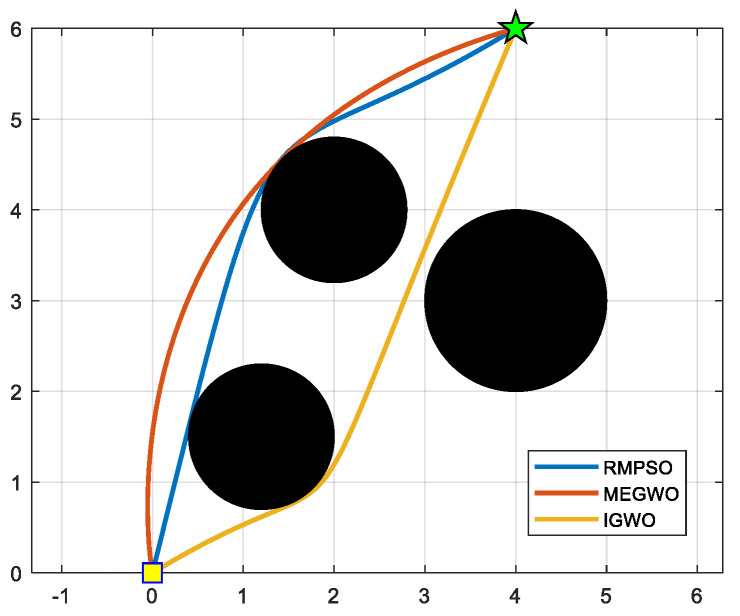
Comparison of three algorithm in case 1, where the yellow square and green five-pointed star respectively represent the starting point and ending point.

**Figure 24:**
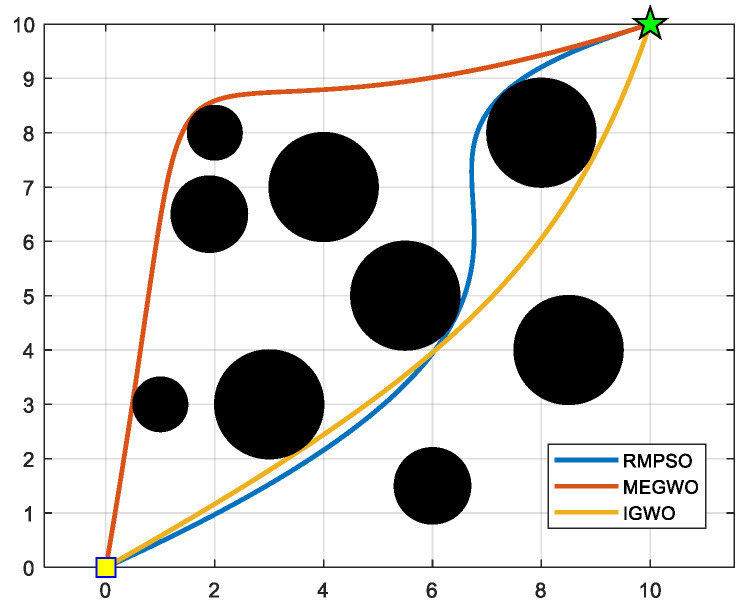
Comparison of three algorithm in case 2, where the yellow square and green five-pointed star respectively represent the starting point and ending point.

**Figure 25:**
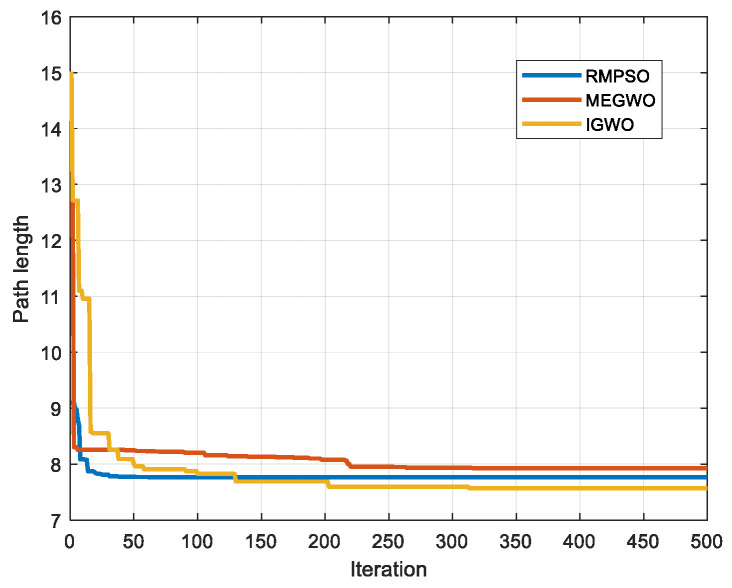
Iteration curves in case 1.

**Figure 26:**
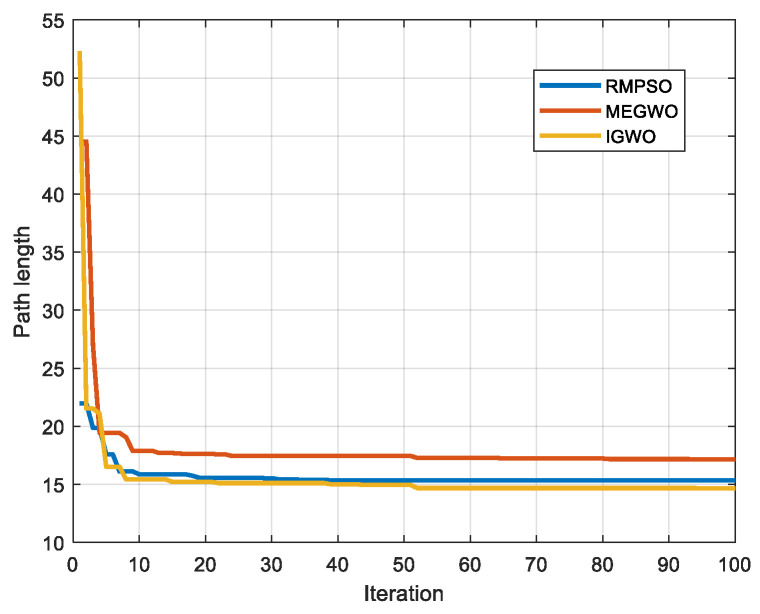
Iteration curves in case 2.

In this subsection, IGWO is applied to robot path planning and compared with MEGWO and RMPSO to verify the superiority of IGWO in solving robot path planning problems. The simulation results of the two cases are shown in Figure 23, Figure 24, Figure 25 and Figure 26. The schematic diagrams of the routes planned by the three algorithms are exhibited in Figure 23 and Figure 24. Figure 25 and Figure 26 show the trend of the path length with the number of iterations.

Figure 23 and Figure 24 indicate that as the environment gets more complex, IGWO generates the path closest to the optimal in both cases. Additionally, Figure 25 demonstrates that IGWO can generate a shorter path compared with RMPSO and MEGWO in case 1. It can be seen from Figure 26 that when the number and density of environmental obstacles are increased, IGWO keep its strengths of the path length. Therefore, the improved algorithm can plan shorter paths in simple and complex environment and has better path optimization performance.

#### 5.5.2. Thirty Independent Contrast Experiments

**Figure 27:**
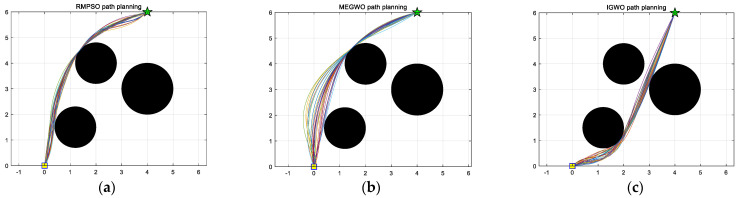
Results of the three algorithms in case 1, where the yellow square, green five-pointed star and lines in different colors in the figure represent the starting point, ending point and paths, respectively. (**a**) Results of RMPSO; (**b**) Results of MEGWO; (**c**) Results of IGWO.

**Figure 28:**
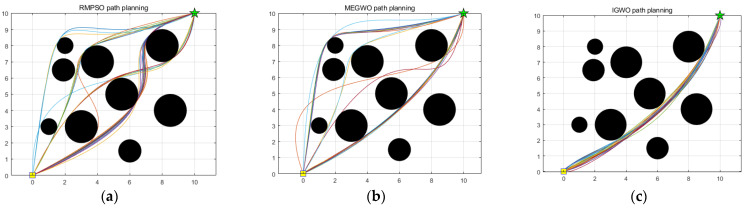
Results of the three algorithms in case 2, where the yellow square, green five-pointed star and lines in different colors in the figure represent the starting point, ending point and paths, respectively (**a**) Results of RMPSO; (**b**) Results of MEGWO; (**c**) Results of IGWO.

**Figure 29:**
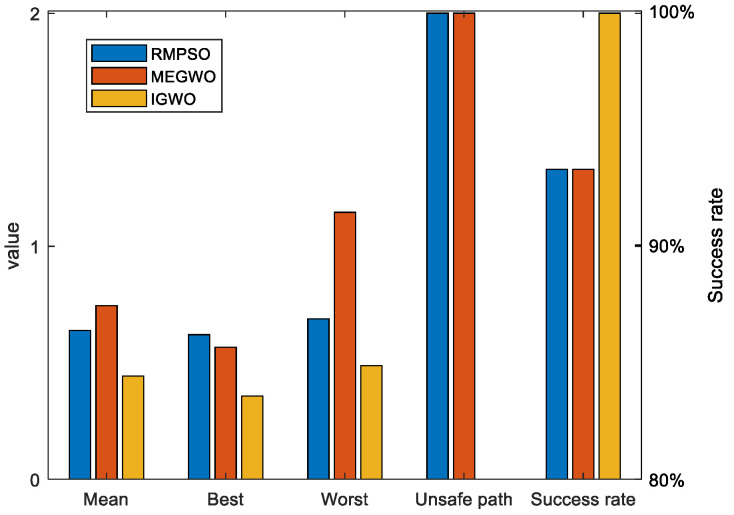
The bar chart of the results in case 1.

**Figure 30:**
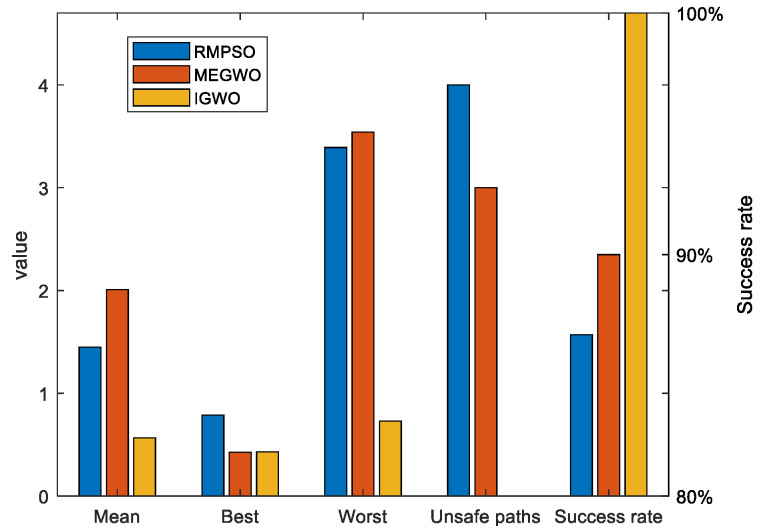
The bar chart of the results in case 2.

**Figure 31:**
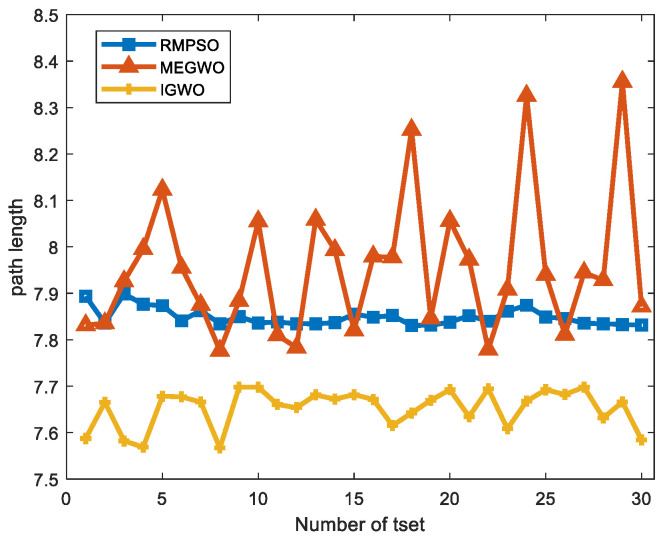
The path length curves in case 1.

**Figure 32:**
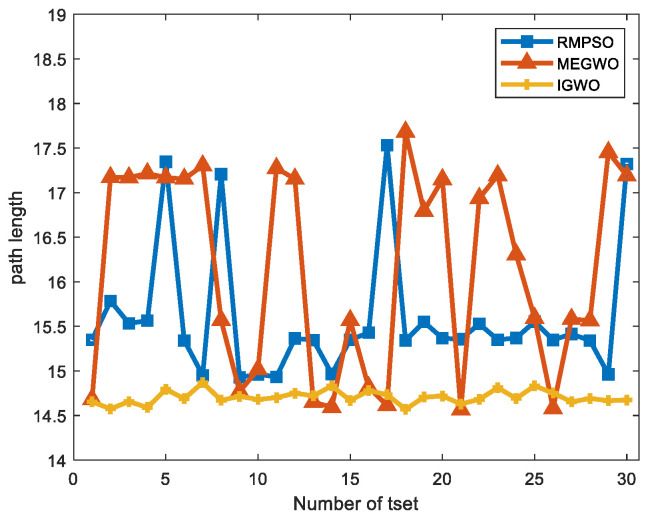
The path length curves in case 2.

Since the results of the single experiment may be accidental, this subsection, we use three algorithms to conduct thirty independent experiments in two obstacle environments, which makes up for the deficiency of the single experiment. Simulation experiment results of path planning in two terrains are shown in Figure 27 and Figure 28. The statistical results are listed in Table 11 and Table 12, where Mean, Best, Worst represent the average, the shortest and the longest length of the thirty planned paths, respectively. Unsafe path represents the number of paths that touch obstacles in the thirty independent tests. Success rate signifies the percentage of secure paths obtained in thirty independent experiments. To visualize the differences between the three algorithms, we convert the experimental results into Figure 29 and Figure 30, where three indexes (Mean, Best, and Worst) are subtracted from the shortest path length listed in Table 9 to observe the gaps with the shortest path more conveniently. Moreover, Figure 31 and Figure 32 exhibit the path length obtained by three methods in thirty experiments, which make it easier to compare the performance of these methods in each experiment.

For the performance of the algorithms in case 1, Figure 27 shows that the planned trajectory of RMPSO and IGWO are relatively stable, while the thirty trajectories of MEGWO fluctuate slightly. Table 11 shows that the average, shortest, and longest length of the paths planned by IGWO are all the smallest among the three algorithms. Furthermore, RMPSO and MEGWO generate three failed paths in the thirty independent experiments, and IGWO plans thirty safe paths successfully, indicating that IGWO possesses better stability. Figure 29 turns the statistical results into a bar graph, more intuitively showing the superiority of IGWO in case 1.

With respect to case 2, Figure 28 denotes that with the increase of obstacles, the gaps between the three algorithms become prominent. The behaviors of RMPSO and MEGWO appear obvious jitter, while IGWO plans the path in more efficient and safer manner. Table 12 illustrates that although MEGWO plans the shortest path, IGWO owns a better performance in the remaining indicators. It is worth mentioning that the success rate of IGWO maintains in 100% despite the increased complexity of the environment, while the success rates of RMPSO and MEGWO are 86.67% and 90% respectively. In addition, the statistical data presented in Figure 30 vividly signifies the better search stability and accuracy of IGWO.

Figure 31 and Figure 32 exhibit the length of thirty paths obtained by each method. It can be observed that there are obvious differences in the performance of the three methods. In case 1, IGWO gets the optimal path in every attempt, and the path length is the most stable, while MEGWO has apparent oscillation. For case 2, IGWO obtains the maximum number of optimal paths compared with RMPSO and MEGWO. Even if the environment complexity is increased, the length of the paths generated by IGWO is stably between 14.5 and 15. In contrast, the path length obtained by RMPSO and MEGWO are sometimes less than 15 and sometimes more than 17. Therefore, it is concluded that the trajectories generated by IGWO are both the shortest and the safest.

### 5.6. Contrast Experiment in Complex Environment with Irregular Obstacles

**Figure 33:**
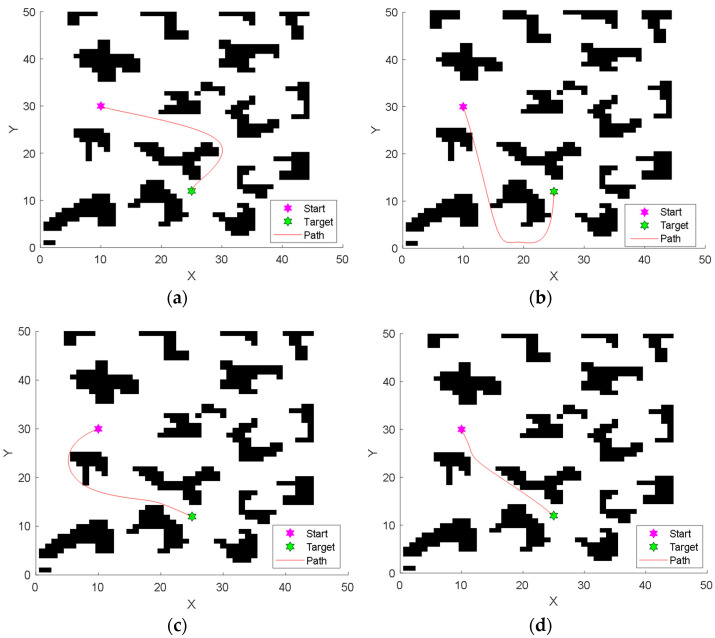
Paths planned by four algorithms in complex obstacle environment. (**a**) Results of RMPSO; (**b**) Results of MEGWO; (**c**) Results of mGWO; (**d**) Results of IGWO.

To further test the capability of the proposed IGWO to solve the path planning problem, a 50 × 50 complex environment model with more irregular obstacles is designed in this section, as shown in Figure 33. Inspired by the polygonal obstacles designed by Dai et al. [64], 18 irregular-shaped obstacles are used in the new environment model. The path starts at (10, 30) and ends at (25, 12).

Beiravand et al. [65] provides the standards and guidelines for researchers to test the performance of optimization algorithms. In this section, comparison experiments are designed referring to [65]. To examine the performance of the proposed IGWO, RMPSO, MEGWO and mGWO are selected as the comparative algorithms. To ensure the fairness and objectivity of the comparison experiments, all experiments are run on CPU Core I5-9400F and 8GB RAM, and programmed on MATLAB R2018b. To avoid randomness of results, all algorithms are run independently for 30 times. Beiravand et al. indicated that algorithm performance metrics can be classified into three categories: efficiency, solution quality and reliability [65]. Therefore, Iteration, Path length and Success Rate are used as performance metrics in this section. The final results are shown in Table 13, with the optimal values among the four algorithms marked in bold. Iteration is the number of iterations when the algorithm obtains the final result, and Path length represents the length of the final path obtained by the algorithm. The values of Iteration and Path length take the average of the results of 30 independent experiments. Success Rate is equal to the ratio of the number of safe paths planned by the algorithm to the total number of independent experiments.

Figure 33 shows the trajectories planned by the four algorithms, visually demonstrating that the proposed IGWO is capable of planning the shorter safe path. Table 13 shows that IGWO achieves the best results in all three metrics compared to the comparative algorithms. In terms of the metric Iteration, the average number of iterations taken by IGWO is 72, which is 24, 21 and 29 generations earlier than RMPSO, MEGWO and mGWO, respectively, indicating that IGWO can search for safe paths more efficiently. In terms of metric Path length, IGWO plans shorter paths among the four algorithms. Since a shorter path length means lower energy consumption, the paths obtained by IGWO are of higher quality. In addition, the planning success rate of IGWO is 90%, ahead of the other algorithms, implying that the IGWO algorithm has higher safety and stability under complex environment model.

## 6. Conclusions and Future Work

In this paper, an improved grey wolf optimizer, namely IGWO, is proposed and applied to robot path planning. The proposed IGWO uses the three well-designed enhancement strategies to balance the global and local search abilities and settle the defects of prematurity and slow convergence to some extent. More exactly, a modified position update mechanism with an improved updating formula is firstly proposed to strengthen the leadership hierarchy of the pack and coordinate the global and local searches more reasonably in the meantime. Simultaneously, the population opposition-based learning and Cauchy mutation are introduced to ensure the diversity of the algorithm population. Then, inspired by the random walk strategy, a dynamic local optimum escape strategy is designed to help the algorithm jump out of the local stagnation. Finally, the bad individual repositioning method is proposed to effectively accelerate the convergence.

IGWO is testified on twenty benchmark functions, and first compared with five state-of-the-art GWO variants, and then seven other well-known meta-heuristic algorithms. Furthermore, Wilcoxon rank-sum test is utilized to judge the significance difference between IGWO and the comparison algorithm. Empirical results show that IGWO outperforms the competitors on most functions. Although IGWO misses the optimal solutions on some functions, by reference to No Free Lunch (NFL), no algorithm can always obtain optimal results in all fields. Afterwards, IGWO is used to deal with robot path planning problems, and cubic spline interpolation is imported to smooth the trajectory of robot. Experimental results reveal that IGWO is a reliable method to solve robot path planning problems, whether in the simple environment or complex environment.

For future work, we will conduct further research in terms of the following issues: First, IGWO will be applied to more challenging terrains, taking the dynamic obstacles into consideration. Second, it’s worth a try to carry out practical experiments to testify the application value and practical significance of the algorithm. Finally, combining GWO with other excellent meta-heuristic algorithms, and the hybrid algorithm may have a more promising performance.

## Figures and Tables

**Table 1:** Unimodal, multimodal and shifted and rotated multimodal benchmark functions. *F* * symbolizes the optimal value for each benchmark function.

Function	Test Function	*Dim*	*Range*	F *
F1	$f_{1}=\sum_{i=1}^{n}x_{i}^{2}$	30	[−100, 100]*^n^*	0
F2	$f_{2}=\sum_{i=1}^{n}\left|x_{i}\right|+\prod_{i=1}^{n}\left|x_{i}\right|$	30	[−10, 10]*^n^*	0
F3	$f_{3}=\sum_{i=1}^{n}\left(\sum_{j=1}^{i}x_{j}\right)$	30	[−100, 100]*^n^*	0
F4	$f_{4}=\underset{n}{\max_{i}}\left\{\left|x_{i}\right|,\;1\leq i\leq n\right\}$	30	[−100, 100]*^n^*	0
F5	$f_{5}=\sum_{i=1}^{n}\left[100{\left(x_{i+1}-x_{i}^{2}\right)}^{2}+{\left(x_{i}-1\right)}^{2}\right]$	30	[−30, 30]*^n^*	0
F6	$f_{6}=\sum_{i=1}^{n}ix_{i}^{4}+random\left[0,\;1\right)$	30	[−1.28, 1.28]*^n^*	0
F7	$f_{7}=\sum_{i=1}^{n}\left[x_{i}^{2}-10\cos\left(2\pi x_{i}\right)+10\right]$	30	[−5.12, 5.12]*^n^*	0
F8	$f_{8}=-20\exp\left(-0.2\sqrt{\frac{1}{n}\sum_{j=1}^{n}x_{j}}\right)-\exp\left(\frac{1}{n}\cos\left(2\pi x_{j}\right)\right)+20+e$	30	[−32, 32]*^n^*	0
F9	$f_{9}=\frac{1}{4000}\sum_{i=1}^{n}x_{i}^{2}-\prod_{i=1}^{n}\cos\left(\frac{x_{i}}{\sqrt{i}}\right)+1$	30	[−600, 600]*^n^*	0
F10	$\begin{array}{l} f_{10}=\frac{\pi }{n}\left\{10\sin\left(\pi y_{1}\right)+\sum_{i=1}^{n}{\left(y_{i}-1\right)}^{2}\left[1+10\sin^{2}\left(\pi y_{i+1}\right)\right]+\sum_{i=1}^{n}u\left(x_{i},\;10,\;100,\;4\right)\right\}\\ y_{i}=1+\frac{x_{i}+1}{4}\\ u\left(x_{i},\;a,\;k,\;m\right)=\left\{ \begin{array}{ll} k{\left(x_{i}-a\right)}^{m} & x_{i}>a\\ 0 & -a<x_{i}<a\;\\ k{\left(-x_{i}-a\right)}^{m} & x_{i}<-a\; \end{array}\right. \end{array}$	30	[−50, 50]*^n^*	0
F11	Shifted and Rotated Katsuura Function	30	[−100, 100]*^n^*	1200
F12	Shifted and Rotated HappyCat Function	30	[−100, 100]*^n^*	1300
F13	Shifted and Rotated HGBat Function	30	[−100, 100]*^n^*	1400

**Table 2:** Fixed-dimension multimodal and composition benchmark functions. *F* * symbolizes the optimal value for each benchmark function.

Function	Test Function	*Dim*	*Range*	F *
F14	$f_{14}={\sum_{i=1}^{11}\left[a_{i}-\frac{x_{i}\left(b_{i}^{2}+b_{i}x_{2}\right)}{b_{i}{}^{2}+b_{i}x_{3}+x_{4}}\right]}^{2}$	4	[−5, 5]*^n^*	0.00030
F15	$f_{15}={\left(x_{2}-\frac{5.1}{4\pi^{2}}x_{1}{}^{2}+\frac{5}{\pi }x_{1}-6\right)}^{2}+10\left(1-\frac{1}{8\pi }\right)\cos\left(x_{1}\right)+10$	2	[−5, 5]*^n^*	0.398
F16	$\begin{array}{l} f_{16}=\left[1+{\left(x_{1}+x_{2}+1\right)}^{2}\left(19-14x_{1}+3x_{1}^{2}-14x_{2}+6x_{1}x_{2}+3x_{2}{}^{2}\right)\right]\\ \times \left[30+{\left(2x_{1}-3x_{2}\right)}^{2}\times \left(18-32x_{1}+12x_{1}{}^{2}+48x_{2}-36x_{1}x_{2}+27x_{2}{}^{2}\right)\right] \end{array}$	2	[−2, 2]*^n^*	3
F17	$f_{17}=-\sum_{i=1}^{5}{\left[\left(X-a_{i}\right){\left(X-a_{i}\right)}^{T}+c_{i}\right]}^{-1}$	4	[0, 10]*^n^*	−10.1532
F18	Composition Function 1 (N = 5)	30	[−100, 100]*^n^*	2300
F19	Composition Function 2 (N = 3)	30	[−100, 100]*^n^*	2400
F20	Composition Function 3 (N = 3)	30	[−100, 100]*^n^*	2500

**Table 3:** Results of GWO variants on unimodal, multimodal and shifted and rotated multimodal benchmark functions. The best values are highlighted in bold.

		MixedGWO	GWOCS	LearnGWO	mGWO	RW_GWO	IGWO
F1	Mean	3.83 × 10^−30^	1.60 × 10^−59^	1.3498 × 10^−123^	7.04 × 10^−60^	1.46 × 10^−22^	**0**
	Std	3.94 × 10^−29^	2.27 × 10^−58^	2.8395 × 10^−122^	1.47 × 10^−58^	9.50 × 10^−22^	**0**
	R/T	5/+	4/+	2/+	3/+	6/+	1
F2	Mean	1.09 × 10^−18^	4.00 × 10^−35^	6.09 × 10^−69^	3.02 × 10^−38^	1.04 × 10^−11^	**0**
	Std	1.02 × 10^−17^	2.50 × 10^−34^	4.57 × 10^−68^	7.56 × 10^−37^	1.96 × 10^−11^	**0**
	R/T	5/+	4/+	2/+	3/+	6/+	1
F3	Mean	8.28 × 10^−5^	3.57 × 10^−15^	2.83 × 10^−89^	3.35 × 10^+3^	1.02 × 10^−9^	**0**
	Std	0.0011	9.19 × 10^−14^	7.08 × 10^−88^	1.23 × 10^+4^	1.41 × 10^−8^	**0**
	R/T	5/+	3/+	2/+	6/+	4/+	1
F4	Mean	7.28 × 10^−6^	1.01 × 10^−14^	2.91 × 10^−51^	3.44 × 10^−10^	5.46 × 10^−8^	**0**
	Std	6.54 × 10^−5^	1.03 × 10^−13^	3.76 × 10^−50^	9.61 × 10^−9^	1.37 × 10^−6^	**0**
	R/T	6/+	3/+	2/+	4/+	5/+	1
F5	Mean	28.8373	26.9401	28.5497	28.6775	**26.5379**	27.5219
	Std	0.1036	3.7477	1.9255	0.7313	**2.9594**	3.7676
	R/T	6/+	2/−	4/+	5/+	1/−	3
F6	Mean	0.0025	8.88 × 10^−4^	**8.10 × 10^−5^**	4.16 × 10^−4^	9.20 × 10^−4^	8.95 × 10^−5^
	Std	0.0051	0.0018	**3.21 × 10^−4^**	0.0021	2.70 × 10^−3^	2.50 × 10^−4^
	R/T	6/+	4/+	1/−	3/+	5/+	2
F7	Mean	19.3405	1.3586	**0**	7.0415	1.4371	**0**
	Std	50.5218	19.1616	**0**	144.5174	17.6327	**0**
	R/T	5/+	2/+	1/≈	4/+	3/+	1
F8	Mean	1.58 × 10^14^	1.55 × 10^−14^	4.56 × 10^−15^	4.56 × 10^−15^	6.18 × 10^−12^	**8.88 × 10^−16^**
	Std	2.87 × 10^−14^	1.58 × 10^−14^	3.49 × 10^−15^	6.12 × 10^−15^	1.19 × 10^−11^	**0**
	R/T	5/+	4/+	2/+	3/+	6/+	1
F9	Mean	0.0021	5.75 × 10^−4^	1.11 × 10^−4^	0.0021	3.26 × 10^−4^	**0**
	Std	0.0409	0.0112	0.0033	0.0632	0.0096	**0**
	R/T	5/+	4/+	2/≈	6/≈	3/+	1
F10	Mean	0.5419	0.0396	0.6079	0.0619	**0.0012**	0.1822
	Std	0.8062	0.1137	0.8432	0.1819	**0.009**	0.5448
	R/T	5/+	2/−	6/+	3/−	1/−	4
F11	Mean	1203.1	1202.5	1203.2	1202.0	**1200.9**	1201.5
	Std	1.4643	7.837	2.5871	1.8016	**5.2961**	2.1432
	R/T	4/+	6/+	5/+	3/+	1/−	2
F12	Mean	1302.1	1300.7	1304.4	1304.0	1300.6	**1300.5**
	Std	6.7783	3.1098	2.5492	4.0168	0.5621	**0.0695**
	R/T	4/+	3/+	6/+	5/+	2/+	1
F13	Mean	1435.3	1410.8	1499.5	1472.9	1400.8	**1400.4**
	Std	107.6842	50.4025	124.8757	126.7876	1.7689	**1.7390**
	R/T	4/+	3/+	6/+	5/+	2/+	1

**Table 4:** Results of GWO variants on fixed-dimension multimodal and composition benchmarks. The best values are highlighted in bold.

		MixedGWO	GWOCS	LearnGWO	mGWO	RW_GWO	IGWO
F14	Mean	0.0099	3.78 × 10^−4^	5.70 × 10^−3^	8.73 × 10^−4^	0.0023	**3.33 × 10^−4^**
	Std	0.0073	0.0013	4.37 × 10^−2^	0.0047	0.0329	**2.58 × 10^−4^**
	R/T	6/+	2/+	4/+	3/+	5/+	1
F15	Mean	0.4576	0.398	0.4003	0.398	0.398	**0.398**
	Std	0	8.74 × 10^−7^	0.0224	3.97 × 10^−8^	2.14 × 10^−6^	**1.03 × 10^−8^**
	R/T	6/+	3/+	5/+	2/+	4/+	1
F16	Mean	**3**	3	3.0093	3	3	3
	Std	**0**	3.69 × 10^−5^	0.273	1.06 × 10^−12^	6.53 × 10^−5^	3.56 × 10^−5^
	R/T	1/−	4/+	6/+	2/−	5/+	3
F17	Mean	**−10.4028**	−9.873	−4.8074	−9.1627	−9.481	−10.057
	Std	**2.83 × 10^−4^**	7.1031	3.8401	12.3134	11.3324	5.7099
	R/T	1−	3/+	6/+	5/+	4/+	2
F18	Mean	2662.4	2642.7	2745.2	2673.7	2626.2	**2500**
	Std	115.9621	69.9378	210.9216	190.9163	23.952	**0**
	R/T	5/+	3/+	4/+	6/+	2/+	1
F19	Mean	2600.2	2600	2600	2600.4	2600	**2600**
	Std	0.3285	0.0604	2.1052 × 10^−6^	1.4886	0.09	**0**
	R/T	5/+	3/+	2/+	6/+	4/+	1
F20	Mean	2704.5	2700.4	2700	2700.9	2712.5	**2700**
	Std	38.3336	13.1324	6.4311 × 10^−13^	27.4906	30.3025	**4.5475 × 10^−13^**
	R/T	5/+	3/+	2/+	4/+	6/+	1

**Table 5:** Overall Wilcoxon rank-sum test results and mean rank results of GWO variants.

Result	MixedGWO	GWOCS	LearnGWO	mGWO	RW_GWO	IGWO
+/≈/−	18/0/2	18/0/2	17/2/1	17/1/2	17/0/3	~
Mean rank	4.7	3.25	3.5	4.05	3.75	1.5
Overall rank	6	2	4	5	3	1

**Table 6:** Results of unimodal, multimodal and shifted and rotated multimodal benchmark functions.

		GWO	SCA	PSO	WOA	ABC	TSA	MVO	IGWO
F1	Mean	2.07 × 10^−59^	1.25 × 10^−2^	9.43 × 10^−9^	6.59 × 10^−150^	0.7887	6.02 × 10^−6^	0.2963	**0**
	Std	1.33 × 10^−58^	1.07 × 10^−1^	1.88 × 10^−7^	1.77 × 10^−148^	2.1909	1.59 × 10^−5^	0.3969	**0**
	R/T	3/+	6/+	4/+	2/+	8/+	5/+	7/+	1
F2	Mean	1.83 × 10^−34^	1.20 × 10^−5^	6.0004	1.27 × 10^−102^	0.0363	0.0208	0.4606	**0**
	Std	1.78 × 10^−33^	1.26 × 10^−5^	38.9865	2.18 × 10^−101^	0.056	0.0305	1.1093	**0**
	R/T	3/+	4/+	8/+	2/+	6/+	5/+	7/+	1
F3	Mean	1.20 × 10^−12^	6.57 × 10^3^	16.3022	1.76 × 10^4^	3.41 × 10^4^	2.67 × 10^4^	45.3638	**0**
	Std	3.44 × 10^−11^	4.86 × 10^3^	46.3105	4.31 × 10^4^	2.04 × 10^4^	1.49 × 10^4^	120.8618	**0**
	R/T	2/+	5/+	3/+	6/+	8/+	7/+	4/+	1
F4	Mean	2.25 × 10^−14^	17.9	0.6072	36.9902	53.3308	26.7721	1.0175	**0**
	Std	1.07 × 10^−13^	9.85	0.6718	113.8156	24.6552	17.5272	1.7441	**0**
	R/T	2/+	5/+	3/+	7/+	8/+	6/+	4/+	1
F5	Mean	**26.7285**	579.7010	56.6336	27.2767	1.72 × 10^4^	125.1067	425.0734	27.6068
	Std	**3.1903**	1.41 × 10^4^	233.9869	3.1856	5.29 × 10^4^	232.5186	3.53 × 10^3^	3.7676
	R/T	1/−	6/+	4/+	2/−	8/+	5/+	7/+	3
F6	Mean	8.28 × 10^−4^	5.09 × 10^−2^	4.8865	0.0021	0.2127	0.2946	0.0222	**9.13 × 10^−5^**
	Std	0.0016	7.87 × 10^−2^	26.4596	0.0104	0.1851	0.2579	0.0465	**2.49 × 10^−4^**
	R/T	2/+	5/+	8/+	3/+	6/+	7/+	4/+	1
F7	Mean	0.7531	10.4	86.7988	8.53 × 10^−15^	224.9901	107.1537	111.7582	**0**
	Std	10.908	16.4	123.4636	1.21 × 10^−13^	93.4056	53.0099	198.3001	**0**
	R/T	3/+	4/+	5/+	2/+	8/+	6/+	7/+	1
F8	Mean	1.67 × 10^−14^	16.1	4.24 × 10^−05^	4.97 × 10^−15^	1.569	1.3307	0.9705	**8.88 × 10^−16^**
	Std	1.55 × 10^−14^	8.48	2.64 × 10^−04^	1.36 × 10^−14^	2.5771	4.6003	3.2009	**0**
	R/T	3/+	8/+	4/+	2/+	7/+	6/+	5/+	1
F9	Mean	0.0036	0.335	0.0094	0.0036	0.8348	0.2145	0.554	**0**
	Std	0.0299	0.335	0.0443	0.0699	0.4122	0.3981	0.5881	**0**
	R/T	2/+	6/+	4/+	3/≈	8/+	5/+	7/+	1
F10	Mean	0.0427	4.1166	0.0173	**0.0069**	6.3958 × 10^3^	0.6925	1.2145	0.1822
	Std	0.1214	68.7625	0.2116	**0.0234**	6.5686 × 10^4^	2.1067	4.9150	0.5448
	R/T	3/−	7/+	2/−	1/−	8/+	5/+	6/+	4
F11	Mean	1202.3	1203.1	**1200.4**	1202.3	1203.3	1201.5	1200.7	1201.5
	Std	6.2334	1.2522	**1.6106**	3.9568	2.4321	2.8913	1.8405	1.1522
	R/T	6/+	7/+	1/−	5/+	8/+	4/+	2/−	3
F12	Mean	1300.6	1303.8	1300.5	1300.6	1300.6	1300.6	1300.7	**1300.5**
	Std	2.0869	1.7531	0.6553	0.7098	0.4312	0.3043	0.8459	**0.0700**
	R/T	6/+	8/+	2/+	5/+	4/+	3/+	7/+	1
F13	Mean	1405.5	1472.1	1400.7	1403.0	1400.8	**1400.3**	1400.7	1400.6
	Std	44.3370	74.1032	8.0383	26.9729	0.2715	**0.2175**	1.9733	1.9105
	R/T	7/+	8/+	4/+	6/+	5/+	1/−	3/+	2

**Table 7:** Results of fixed-dimension multimodal and composition benchmark functions.

		GWO	SCA	PSO	WOA	ABC	TSA	MVO	IGWO
F14	mean	0.0014	1.00 × 10^−3^	0.0057	6.09 × 10^−4^	6.22 × 10^−4^	3.83 × 10^−4^	0.0034	**3.33 × 10^−4^**
	sd	0.0195	4.23 × 10^−4^	0.0335	0.0015	4.51 × 10^−4^	2.51 × 10^−4^	0.0366	**2.58 × 10^−4^**
	R/T	6/+	5/+	8/+	3/+	4/+	2/+	7/+	1
F15	mean	0.398	0.399	**0.398**	0.398	**0.398**	**0.398**	0.398	0.398
	sd	9.97 × 10^−5^	1.45 × 10^−3^	**0**	2.43 × 10^−6^	**0**	**0**	1.16 × 10^−6^	5.71 × 10^−08^
	R/T	5/+	6/+	1/−	4/+	1/−	1/−	3/+	2
F16	mean	3	3	3	3	**3**	3	5.7	3
	sd	2.95 × 10^−05^	1.37 × 10^−05^	4.97 × 10^−15^	1.50 × 10^−04^	**1.54 × 10^−15^**	3.29 × 10^−15^	79.6386	1.07 × 10^−06^
	R/T	6/+	5/+	3/−	7/+	1/−	2/−	8/+	4
F17	mean	−9.8160	−3.45	−7.9464	−8.5398	−10.1526	**−10.1532**	−7.6246	−10.0786
	sd	6.9026	12.5768	15.1201	13.6539	0.0175	**1.46 × 10^−14^**	15.1507	2.3102
	R/T	4/+	8/+	7/+	5/+	2/−	1/−	6/+	3
F18	mean	2641.6	2713.4	2616.4	2668.3	2617.4	2615.3	2623.7	**2500**
	sd	68.8862	142.2616	12.1211	264.5586	4.1657	0.0414	30.339	**0**
	R/T	6/+	8/+	3/+	7/+	4/+	2/+	5/+	1
F19	mean	2600	2607.1	2624.4	2608.7	2638.5	2633.5	2636.5	**2600**
	sd	0.0727	40.0755	43.338	35.6168	20.2054	8.637	35.5488	**0**
	R/T	2/+	3/+	5/+	4/+	8/+	6/+	7/+	1
F20	mean	2712.3	2739.6	2718.1	2722.9	2741.4	2719.9	2708.1	**2700**
	sd	31.8856	50.1478	26.4854	112.5271	42.1794	13.3521	11.7235	**4.55 × 10^−13^**
	R/T	3/+	7/+	4/+	6/+	8/+	5/+	2/+	1

**Table 8:** Overall Wilcoxon rank-sum test results and mean rank results.

Result	GWO	SCA	PSO	WOA	ABC	TSA	MVO	IGWO
+/≈/−	18/0/2	20/0/0	16/0/4	17/1/2	17/0/3	16/0/4	19/0/1	~
Mean rank	3.75	6.05	4.15	4.1	6	4.2	5.4	1.7
Overall rank	2	8	4	3	7	5	6	1

**Table 9:** Environmental model information.

	Simple Environment (Case 1)	Complex Environment (Case 2)
Obstacles	3	9
Starting point	(0,0)	(0, 0)
Ending point	(4,6)	(10, 10)
The shortest length	7.21	14.14

**Table 10:** Experimental parameter configuration.

	Simple Environment (Case 1)	Complex Environment (Case 2)
Population size	30	30
Path points	2	2
Interpolation points	100	100
Iterations	100	100

**Table 11:** Results comparison in case 1, where the best values are highlighted in bold.

	Mean	Best	Worst	Unsafe Path	Success Rate
RMPSO	7.8486	7.8308	7.8989	3	90%
MEGWO	7.9557	7.7764	8.3557	3	90%
IGWO	**7.6529**	**7.5669**	**7.6981**	**0**	**100%**

**Table 12:** Results comparison in case 2, where the best values are highlighted in bold.

	Mean	Best	Worst	Unsafe Paths	Success Rate
RMPSO	15.5894	14.9284	17.5323	4	86.67%
MEGWO	16.1491	**14.5662**	17.6817	3	90%
IGWO	**14.7052**	14.5691	**14.8698**	**0**	**100%**

**Table 13:** Experimental results in complex obstacle environment, and the best values are highlighted in bold.

	Iteration	Path Length	Success Rate
RMPSO	96	36.289	86.67%
MEGWO	93	40.1963	83.33%
mGWO	101	39.3321	80%
IGWO	**72**	**31.8779**	**90%**

## Data Availability

Not applicable.
